# Integrating cells, scaffolds, and molecular regulation: a mechanobiological and translational review of bioengineering therapies for intervertebral disc degeneration

**DOI:** 10.3389/fbioe.2026.1803183

**Published:** 2026-04-17

**Authors:** Wang Hao, Chen Renchang, Xia Wa, Huang Wenhao, Zhou Bingqian, Zheng Xiqiu, Wang Jiahao, Wu Yadong, Li Nianhu

**Affiliations:** 1 First Clinical Medical College, Shandong University of Traditional Chinese Medicine, Jinan, China; 2 Department of Emergency Surgery, Danyang Traditional Chinese Medicine Hospital, Zhenjiang, China; 3 Department of Spine Orthopedics, Rizhao Hospital of Traditional Chinese Medicine, Rizhao, China; 4 Department of Spine and Spinal Cord Surgery, The Affiliated Hospital of Shandong University of Traditional Chinese Medicine, Jinan, China

**Keywords:** bioengineering, biomaterials, cell therapy, gene therapy, intervertebral disc degeneration, mechanobiology, tissue engineering

## Abstract

Intervertebral disc degeneration (IDD) is a primary cause of chronic low back pain, severely impacting patients' quality of life. Conventional treatments focus on symptom relief but fail to restore disc structure and function. Recent bioengineering advances offer regenerative solutions, integrating cell therapy, tissue-engineered scaffolds, gene therapy, and mechanobiology. Cell therapy leverages mesenchymal stem cells (MSCs) from bone marrow, adipose tissue, or umbilical cord blood, with biomaterial carriers enhancing survival in the harsh disc microenvironment. Scaffolds—natural (collagen, chitosan) or synthetic (PLGA, PCL)—mimic native extracellular matrix (ECM) and provide mechanical support, often combined with growth factors for controlled release. Gene therapy targets ECM synthesis, inflammation, and degradation pathways via viral or non-viral vectors, while mechanobiology reveals how mechanical forces regulate disc cell behavior, guiding scaffold design. Animal models validate these therapies, and early clinical trials show promise in pain reduction and disc height restoration. However, challenges remain, including low cell survival, scaffold mechanical adaptation, and gene delivery safety. Multidisciplinary collaboration is key to translating preclinical progress into effective clinical interventions, addressing the unmet medical need for IDD treatment.

## 1 Introduction

Intervertebral disc degeneration is a key part of spinal degenerative diseases. It is the main cause of low back pain (LBP) worldwide. LBP not only reduces quality of life but also brings heavy socioeconomic burdens. IDD involves complex pathological processes. These include disc cell dysfunction, extracellular matrix (ECM) degradation, structural damage, and inflammation. Together, these changes lead to disc degeneration and associated pain (Zhang X. et al., 2023; Devkota et al., 2025).

The intervertebral disc (IDD) is a specialized fibrocartilaginous structure. It has three parts: inner nucleus pulposus (NP), outer annulus fibrosus (AF), and cartilage endplates (CEP). Each part contributes to load-bearing and shock absorption. Degeneration changes the ECM and structure. This impairs the disc’s integrity and function.

Traditional IDD treatments focus on symptom control. Conservative measures and surgeries like discectomy or spinal fusion are common. But these methods do not address the root cause of degeneration. They rarely restore the disc’s natural structure and biomechanics (Nakielski et al., 2023; Liu et al., 2026) Surgery also cannot stop further degeneration or fully restore function. There’s an urgent need for innovative therapies. These therapies should regenerate or repair degenerated disc tissue. They need to restore biomechanical function and relieve pain (Keshavarz et al., 2025).

Recent advances in bioengineering and regenerative medicine have opened new doors. They combine cell biology, materials science, and mechanical engineering. Researchers have developed biomaterials like hydrogels. These mimic the native ECM and support cell survival and tissue regeneration (Wang Y. et al., 2021; Chen T. et al., 2025). Exosome-based therapies also show promise. Stem cells can promote extracellular matrix repair and reduce nucleus pulposus cell apoptosis (Li Z.-P. et al., 2025). Key signaling pathways like mTOR and Wnt/β-catenin are potential targets. mTOR signaling regulates intervertebral disc cell autophagy, oxidative stress, inflammatory response, ECM homeostasis, cell senescence and apoptosis (Chen H.-W. et al., 2023).

Mechanobiology plays a crucial role in IDD’s onset and progression. Abnormal mechanical loading disrupts disc balance. It causes inflammation, oxidative stress, and matrix degradation. Understanding how mechanical factors affect disc cells is vital. It helps design effective regenerative therapies (Neidlinger-Wilke et al., 2024; Qiao et al., 2025). Advanced imaging like quantitative MRI aids diagnosis. It evaluates degeneration severity and microenvironmental changes non-invasively (Ji et al., 2020).

Animal models and *in vitro* systems have been key to research. Models like needle puncture or mechanical overload replicate human IDD. Intervertebral disc degeneration animal models allow researchers to study the natural history of intervertebral disc degeneration and identify therapeutic targets (Liang et al., 2022). But translating findings to humans is challenging. Human disc anatomy, physiology, and pathology are more complex.

In short, the synergistic application of mesenchymal stem cells, their extracellular vesicles and hydrogel scaffolds holds great promise for IDD regenerative treatment, demonstrating the potential to restore native disc structure and function (Zhao et al., 2024). Unlike most existing reviews, which focus on fragmented advances of individual therapeutic modalities and rarely build a unified logical framework across pathology, therapeutic design and clinical translation, this work takes mechanobiology—a core driver of IDD progression—as the central thread to systematically elaborate the design principles and synergistic mechanisms of cell therapy, engineered scaffolds and gene therapy, establishes a quantitative evaluation system for the “cell-scaffold-molecular regulation” tripartite synergistic strategy. On this basis, we systematically sort out the latest progress of IDD bioengineering therapies, with a focus on preclinical model validation, clinical trial advances and unresolved bench-to-bedside translation challenges.

## 2 Pathological mechanisms of intervertebral disc degeneration and theoretical basis of bioengineering treatment

### 2.1 Overview of intervertebral disc structure and function

The intervertebral disc sits between adjacent vertebrae. It is critical for spinal biomechanics. It bears compressive loads and enables flexibility and movement. Its main components are the nucleus pulposus and annulus fibrosus (Ashinsky et al., 2021; Mirzaeipoueinak et al., 2023).

The NP is a gelatinous core. It is rich in proteoglycans and water. This gives it hydrostatic properties for load distribution. The AF surrounds the NP. It has concentric collagen fiber layers arranged at an angle. This provides tensile strength and contains the NP. The CEP act as a biomechanical interface. They support nutrition and function of the nucleus pulposus and annulus fibrosus (Velnar and Gradisnik, 2023).

The disc’s tissue composition is highly specialized. The matrix is mostly collagen, proteoglycans, and water. Resident cells maintain this matrix (Neidlinger-Wilke et al., 2024). Collagen type I is abundant in the AF. Collagen type II and proteoglycans dominate the NP. These components give the disc its biomechanical properties.

The disc’s function comes from its composite structure. The NP’s pressure resists compression. The AF’s fibers resist circumferential and radial stress during movement. Recent studies highlight the transition zone (TZ) between NP and AF. It ensures structural integration and mechanical continuity. The TZ has unique mechanical properties. It differs in stiffness and energy absorption from NP and AF (Mirzaeipoueinak et al., 2023). Fibers in the TZ adapt, penetrate, and entangle. This maintains stability under physiological loading. The TZ is vital for disc integrity. It may be a key target for therapeutic strategies.

The disc’s function is closely linked to cellular activity. Disc cells include NPCs, AF cells, and endplate chondrocytes. They synthesize and maintain the ECM. They also regulate nutrition in the avascular disc (Zhou H. et al., 2024). These cells adapt to a harsh environment. It is hypoxic, acidic, and low in nutrients. Mitochondria play an important role in the physiology of intervertebral disc and knee articular cartilage tissue, and OPA1 protects tissue health by maintaining mitochondrial structure and metabolic function (Madhu et al., 2024). Mitochondrial dysfunction and oxidative stress contribute to degeneration. This shows cellular health is key to disc function.

Age and degeneration alter the disc’s biomechanics. ECM composition and organization change. This leads to reduced disc height, less hydration, and impaired mechanical properties (Ashinsky et al., 2021). Degeneration affects the disc’s viscoelasticity. It lowers initial modulus and pressure (Sun et al., 2023). Understanding normal structure-function relationships is essential. It helps develop regenerative therapies and biomaterials (Ogaili et al., 2025).

In summary, the intervertebral disc is a specialized structure. Its components and cellular interactions enable load-bearing and flexibility. Advances in understanding its microstructure and biomechanics inform treatment. These findings emphasize the disc as an integrated system. Mechanical, biochemical, and cellular factors work together to maintain function.

### 2.2 Molecular and cellular mechanisms of intervertebral disc degeneration

Intervertebral disc degeneration involves complex molecular and cellular changes. These disrupt disc balance and function. A key feature is increased apoptosis of nucleus pulposus cells (NPCs). Fewer viable cells impair matrix synthesis. This worsens the extracellular matrix, critical for disc integrity (Li et al., 2024d).

Matrix metalloproteinases (MMPs) are upregulated in degenerated discs. These enzymes break down key ECM components. Collagen type II and aggrecan are major targets. Inflammatory cytokines like TNF-α and IL-1β promote this process. The imbalance between matrix synthesis and degradation reduces disc height. It also causes biomechanical dysfunction.

Mechanical stress strongly influences disc cell metabolism. Chronic or excessive loading changes NPC and AFC behavior. It promotes a catabolic state. Cells produce more inflammatory mediators and proteolytic enzymes (Liu F. et al., 2024). Mechanical stress triggers signaling pathways. These increase MMP and pro-inflammatory cytokine expression. This accelerates ECM degradation and cell apoptosis.

Oxidative stress damages disc cells. It causes mitochondrial dysfunction and reactive oxygen species (ROS) buildup. This promotes cell apoptosis and accelerates extracellular matrix degradation (Hao et al., 2026). These stressors create a cycle that worsens degeneration. NPC phenotype changes during degeneration. This impairs biomechanical properties and regenerative capacity. Gene expression shifts: anabolic genes like collagen II are downregulated. Fibrotic markers are upregulated.

Inflammation is closely tied to IDD pathology. Immune cells like macrophages infiltrate degenerated discs. They polarize into pro-inflammatory (M1) or anti-inflammatory (M2) types. This influences disease progression. Macrophage migration inhibitory factor (MIF) is a key mediator. It activates the NF-κB pathway. This increases inflammatory cytokines and matrix breakdown. Senescent NPCs secrete SASP factors. These create a pro-inflammatory environment. They release cytokines and matrix-degrading enzymes (Silwal et al., 2023). Inflammation and senescence together speed up degeneration. Bacterial factors like Propionibacterium acnes may also contribute. Propionibacterium acnes induces intervertebral disc degeneration through mechanisms related to inflammation, pyroptosis and apoptosis (Yang W. et al., 2025). Targeting inflammation and immune cell infiltration could be therapeutic.

Mitochondrial dysfunction is a critical mechanism. Mitochondria regulate energy metabolism, ROS, and apoptosis in NPCs. Impaired mitochondrial dynamics cause problems. Excessive fission and defective mitophagy lead to dysfunctional mitochondria. Oxidative stress and cell death are among the pathogenic mechanisms of intervertebral disc degeneration, and mitochondrial dysfunction can promote the progression of intervertebral disc degeneration through pathophysiological processes including cell death (Song et al., 2023; Hao et al., 2026). Restoring mitochondrial function may help. Strategies like enhancing mitophagy via the HIF-1α-BNIP3-LC3B axis show promise (Chen X. et al., 2025).

In summary, IDD is driven by multiple factors. These include NPC apoptosis, MMP upregulation, inflammatory catabolism, mechanical stress, and mitochondrial dysfunction. NPC phenotypic changes further compromise the disc. Understanding these mechanisms guides targeted interventions. Future research should explore molecular crosstalk. It should validate targets in different IDD subtypes for better translation.

### 2.3 Theoretical basis of bioengineering intervention in intervertebral disc degeneration

**FIGURE 1:**
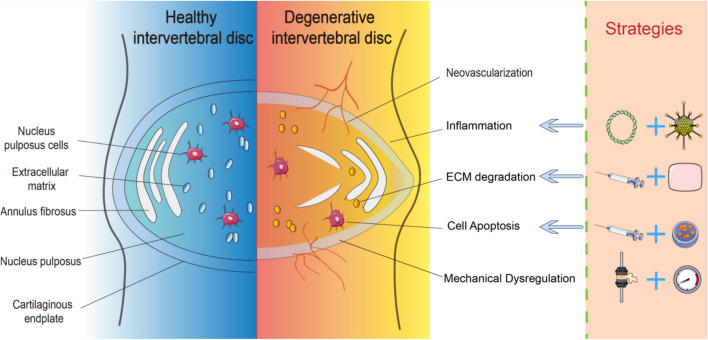
Healthy versus degenerative intervertebral disc: key pathological alterations and targeted therapeutic interventions.

Biomedical engineering aims to restore the structure and function of the intervertebral disc, targeting the pathological pillars: cell replacement, extracellular matrix reconstruction, and mechanical environment regulation. Biomedical engineering therapeutic strategies are built on a unified theoretical framework of the “cell-scaffold-molecular regulation” tripartite synergy: cell therapy replenishes the functional cell pool lost due to degeneration; tissue engineering scaffolds reconstruct the natural extracellular matrix microenvironment and biomechanical support; targeted delivery systems of bioactive factors or gene carriers achieve precise molecular regulation of the degenerative microenvironment (see Figure 1 for details).

Cell replacement therapy commonly uses mesenchymal stem cells (MSCs) or nucleus pulposus mesenchymal stem cells (NPMSCs), which have the potential for intervertebral disc regeneration (Zhang J. et al., 2022). However, the microenvironment of the degenerative intervertebral disc is harsh, which threatens the survival and function of implanted mesenchymal stem cells (Zhang J. et al., 2022).

Tissue engineering combines cell therapy with biomaterial scaffolds, which can simulate the natural ECM, provide physical support and biochemical signals, and enhance cell activity and differentiation ability. Alginate, gelatin methacryloyl (GelMA), and collagen-hyaluronic acid composites have been widely used in the field of intervertebral disc regeneration. This is mainly due to their ability to simulate the viscoelastic characteristics of the nucleus pulposus (NP) and act as delivery carriers for cells and bioactive factors. For example, alginate hydrogels can deliver growth factors or cells for the treatment of intervertebral disc degeneration (Jarrah et al., 2023). Similarly, GelMA hydrogel composite systems have application potential in the treatment of intervertebral disc degeneration (Li et al., 2023). In addition, collagen-hyaluronic acid hydrogels embedded with β-cyclodextrin-resveratrol complexes exhibit inflammation-sensitive drug release properties *in vivo* and can promote nucleus pulposus regeneration (Wang Z. et al., 2025). In summary, these adjustable hydrogel systems can not only replicate the natural biomechanical environment of the nucleus pulposus but also provide targeted delivery therapy, offering promising strategies for intervertebral disc repair and reversal of degeneration.

The intervertebral disc bears complex loads, and degeneration alters its biomechanical behavior, with abnormal swelling pressure and viscoelastic response (Sun et al., 2023). Biomedical engineering constructs aim to restore mechanical function, and scaffolds are designed to have suitable stiffness and resilience to effectively absorb and distribute mechanical loads. The design of biomedical engineering constructs aims to restore the biomechanical function of the intervertebral disc, and their scaffolds are engineered to have suitable stiffness and resilience, thereby effectively absorbing and dispersing mechanical loads. Against this background, electrospun nanofiber scaffolds have become a promising strategy for repairing the annulus fibrosus (AF) and achieving intervertebral disc regeneration. By bionically replicating the anisotropic fiber arrangement of natural AF, these scaffolds can provide structural reinforcement and promote tissue integration. For example, a study designed a biodegradable nanofiber scaffold using poly (ε-caprolactone-co-lactide) (PCLA) with adjustable PLA content (10%–30%), and *in vitro* experiments proved that the scaffold can support the colonization of sheep AF cells and improve their metabolic activity. Electrospun nanofiber-based scaffolds for intervertebral disc repair have made relevant research progress (Li C. et al., 2022). It can be seen that electrospun nanofiber scaffolds are an example of biomedical engineering constructs, which demonstrate how to simulate the mechanical properties of natural tissues through precise design, while providing a supportive microenvironment for cell activities and defect stabilization, thus providing a translatable platform for intervertebral disc regeneration.

Injectable self-healing hydrogels can achieve minimally invasive delivery and adapt to the dynamic mechanical environment of the intervertebral disc (Gu et al., 2025). Nucleus pulposus tissue engineering is a biological therapy that combines histology and materials science (Li Q. et al., 2024; Liu et al., 2026).

Integrating bioactive molecules such as growth differentiation factor 5 (GDF-5) into delivery systems has been shown to significantly enhance the efficacy of intervertebral disc regeneration strategies by promoting cell proliferation and extracellular matrix synthesis. A thermosensitive injectable hydrogel system, which is formed by crosslinking chondroitin sulfate grafted with poly N-isopropylacrylamide and sodium alginate microspheres (HMs), is used for local delivery of GDF-5. *In vitro* experiments show that GDF-5-loaded hydrogels (1 mg/mL) can moderately enhance the proliferation of nucleus pulposus cells (NPCs) and reduce the expression of inflammatory cytokines (TNF-α, IL-6, IL-1β). In addition, *in vivo* evaluation in a rat model of intervertebral disc degeneration showed that the combined delivery of HMs-GDF-5 and adipose-derived mesenchymal stem cells (ADMSCs) to NP tissue through minimally invasive technology can promote significant intervertebral disc regeneration. The core results observed after 8 weeks of treatment include: upregulation of COL-II and Aggrecan (ACAN) protein and their mRNA expression, recovery of intervertebral disc height, increase in water content, and partial recovery of NPC and matrix structure (Chen G. et al., 2025).

The theoretical basis of biomedical engineering stems from the understanding of intervertebral disc pathophysiology, integrating stem cell biology, biomaterials science, and mechanobiology. Future directions include personalized scaffolds customized according to the severity of degeneration and the patient’s biomechanical characteristics; self-powered piezoelectric materials can also be explored to regulate cell behavior through electrical stimulation (Isa et al., 2022; Huang et al., 2024). These innovations could translate bioengineering into clinical therapies.

## 3 Progress in the application of cell therapy in intervertebral disc degeneration

### 3.1 Intervertebral disc cells and their phenotypic characteristics

The intervertebral disc has two main cell populations. Nucleus pulposus cells and annulus fibrosus cells each have unique roles. NPCs are in the gelatinous core. They have a rounded, chondrocyte-like shape. They produce a proteoglycan-rich ECM. This maintains hydration and mechanical resilience. AF cells are in the outer fibrous ring. They are more fibroblast-like. They synthesize collagen fibers for tensile strength.

Maintaining these cell phenotypes is key to disc health. Intrinsic signaling pathways play a role. Hedgehog-Gli3 signaling preserves AF and CEP cell phenotypes. This prevents degeneration (Zhang et al., 2024). Cellular senescence and inflammation disrupt NPC function. Senescent cells secrete SASP factors. These promote catabolic and inflammatory cascades. Senescence-associated secretory phenotype constructs an inflammatory and catabolic environment, affects tissue microstructure, and hinders intervertebral disc homeostasis and tissue regeneration (Liu Y. et al., 2024).

External stimuli influence NPC phenotypic plasticity. Hypoxic priming and ECM scaffolds can help. They restore discogenic markers like SOX9 and FOXO3a. Appropriate stimulation can promote the anabolic activity of degenerated intervertebral disc cells (Penolazzi et al., 2022). Modulating the microenvironment may reverse degenerative phenotypes. This promotes tissue repair.

Mesenchymal stem cells and NPMSCs are promising for regeneration. They have multipotent differentiation and paracrine effects. NPMSCs are adapted to the disc’s harsh environment. High osmolarity is common there (Zhang H. et al., 2022). Systemic metabolic conditions can affect MSC viability and differentiation.

Extracellular vesicles (EVs) from MSCs mediate intercellular communication. They modulate NPC phenotype and promote ECM synthesis. Senolytic agents like o-Vanillin enhance EV release and uptake. o-Vanillin regulates crosstalk between MSCs and disc cells (Li L. et al., 2022). Bioengineered hydrogels mimic the disc’s negatively charged ECM. They are being studied for their effects on NPCs (Bermudez-Lekerika et al., 2024). Researchers combined such hydrogels with growth factors such as GDF5 in SD rat models. Composite hydrogels loaded with GDF5 microspheres combined with nucleus pulposus stem cells can better promote the differentiation of nucleus pulposus stem cells and delay intervertebral disc degeneration compared with other groups (Ma T. et al., 2024).

Researchers have identified NPC subpopulations. Markers like CD24 and GD2 distinguish them. CD24^+^ NPCs retain a more notochordal-like phenotype. Studying the characteristics of different cell subsets in the nucleus pulposus, especially CD24-positive cells, and their role in nucleus pulposus homeostasis (Ionescu et al., 2024). Single-cell RNA sequencing reveals dynamic cell changes post-injury. This includes distinct MSC populations. These findings may guide repair strategies (Clayton et al., 2025a; Clayton et al., 2025b)

In summary, NP and AF cell phenotypes are fundamental to disc integrity. Their dysfunction contributes to degeneration. Stem cells, especially NP-derived progenitors and MSCs, show potential. The disc microenvironment and systemic factors affect their efficacy. Advances in cell subpopulations and biomaterials offer new therapeutic avenues.

### 3.2 Stem cell sources and their induced differentiation

Selecting a suitable stem cell source is a core prerequisite for regenerative therapy of intervertebral disc degeneration (IDD). Currently, bone marrow mesenchymal stem cells (BMSCs), adipose-derived mesenchymal stem cells (ADSCs), and umbilical cord mesenchymal stem cells (UC-MSCs) have become research hotspots due to the convenience of sample collection and their ability to differentiate into nucleus pulposus-like cells.

Among them, it has been verified by *in vitro* non-contact co-culture experiments that hBMSCs and hADSCs can differentiate into hNPCs-like cells by themselves; animal experiments further confirm that transplantation of both can significantly reduce the progression of needle-induced rat caudal IDD degeneration, and both can exert therapeutic effects by delaying the deterioration of degeneration (Zhang J. et al., 2023).

Adipose-derived mesenchymal stem cells show unique advantages in IDD regeneration: donor matching studies show that human adipose-derived mesenchymal stem cells (hADSCs) have significantly better responsiveness to recombinant human growth differentiation factor 6 (rhGDF6) than bone marrow mesenchymal stem cells, and can highly express nucleus pulposus (NP) marker genes and synthesize more proteoglycan-rich matrix (Hodgkinson et al., 2020). This advantage stems from the high expression and low variability of bone morphogenetic protein receptor 2 (BMPR2) in hADSCs, which in turn enhances the activation of downstream SMAD1/5/8 and ERK1/2 signaling pathways; inhibitor experiments have confirmed that SMAD1/5/8 signaling is an essential pathway for rhGDF6 to induce hADSCs to differentiate into nucleus pulposus-like cells, while ERK1/2 regulates the expression of key nucleus pulposus molecules such as ACAN and COL2, providing translational research basis for the selection of cell therapy targets (Hodgkinson et al., 2020).

Umbilical cord mesenchymal stem cells (UC-MSCs) have become another important candidate due to their low immunogenicity and low ethical controversy in sample collection. Studies have shown that human umbilical cord mesenchymal stem cells (hUC-MSCs) can be directed differentiated into chondroprogenitor cells in chondrogenic induction medium (Ekram et al., 2021). In *in vivo* transplantation experiments, compared with normal BMSCs, chondroprogenitor cells derived from hUC-MSCs show better survival, homing, and distribution capabilities in a rat degenerative IDD model, and can significantly regulate pain and inflammation-related gene expression on the fifth day after transplantation, and fluorescence co-localization of DiI-labeled cells with SOX9 and TGF-β1/2 confirms that they can differentiate into functional nucleus pulposus cells. Alcian blue and HE staining results further verify that the IDD regeneration potential of human umbilical cord mesenchymal stem cell-derived chondroprogenitors is superior to that of common human umbilical cord mesenchymal stem cells (Ekram et al., 2021).

Human induced pluripotent stem cells (hiPSCs) provide a “customized” cell source for IDD treatment, and can be induced to generate notochord-like cells (embryonic precursor cells of nucleus pulposus cells) by regulating signaling pathways and epigenetic factors. For example, combined regulation of signaling pathways such as BMP, FGF, retinoic acid, and Wnt, or targeted regulation of epigenetic modifiers such as KDM2A and KDM7A/B, can achieve the directed differentiation of hiPSCs into stable nucleus pulposus-like cells [65], providing new ideas for solving the problems of insufficient stem cell sources and immune rejection.

GDF5 and GDF6 are key regulatory factors for the differentiation of nucleus pulposus-like cells. In related studies on GDF6, in addition to clarifying the response mechanism of hADSCs to GDF6 (Hodgkinson et al., 2020) studies have also found through transcriptome analysis that GDF6 treatment can make the transcription factor ERG1 in hADSCs a key early response gene, and can regulate the expression of related genes to inhibit osteogenic and adipogenic differentiation, thereby promoting the differentiation directionality of hADSCs into nucleus pulposus-like cells (Gilbert et al., 2024). This finding has been verified by gene knockdown and overexpression experiments in in vitro cell models, and in a rat caudal IDD degeneration model. However, this research is still in the basic experimental stage and has not yet entered clinical trials or translational research stages.

Translational research on GDF5 focuses on the optimization of “factor delivery systems” to solve the problems of its short *in vivo* half-life and insufficient local concentration. For example, acellular nucleus pulposus matrix/chitosan hydrogels loaded with GDF5 microspheres are used to construct a “sustained release-colonization” dual-functional delivery system. In a rat IDD degeneration model, this system significantly improved the repair degree of nucleus pulposus tissue compared with the BMSCs combined composite hydrogel group (Ma T. et al., 2024) another study compounded hiPSCs overexpressing GDF5 with thermosensitive hydrogels to construct a local delivery system of cells and factors. In a rat IDD degeneration model, this system can improve nucleus pulposus degeneration, and related indicators are better than the simple hydrogel group and the untreated group (Hu et al., 2020). This study verified the effectiveness of the delivery system through imaging, histological, and molecular biological means, providing technical support for further basic research on GDF5. However, most of the research on GDF5 remains in the basic experimental stage of human cells or animal models, and has not yet entered clinical trials or translational research stages.

The efficient differentiation of stem cells into nucleus pulposus-like cells also depends on microenvironment regulation and molecular mechanism optimization. In terms of the microenvironment, the unique hypoxic microenvironment of the intervertebral disc can mediate protective autophagy of nucleus pulposus-derived stem cells by activating hypoxia-inducible factor 1α (HIF1A), significantly improving their survival rate under mechanical stress (He et al., 2021).

At the molecular mechanism level, non-coding RNA and exosome regulation have become new research directions. For example, miR-140-3p can affect the proliferation, migration, differentiation, and apoptosis processes of degenerative nucleus pulposus-derived mesenchymal stem cells by targeting and regulating KLF5-related pathways. In a rat IDD degeneration model, overexpression of miR-140-3p can alleviate the nucleus pulposus degeneration process (Wang Z. et al., 2021) in addition, urine-derived stem cell exosomes can activate the TGF-β/SMAD and AKT signaling pathways by delivering MATN3, and can promote NPC proliferation and ECM synthesis in in vitro experiments, providing a new research direction for cell-free treatment strategies (Guo et al., 2021).

In summary, bone marrow, adipose, and umbilical cord-derived mesenchymal stem cells and human induced pluripotent stem cells have been confirmed to have IDD treatment potential in cell experiments and animal models, and technologies such as optimization of delivery systems for growth factors such as GDF5/GDF6, simulation of hypoxic microenvironment, and non-coding RNA regulation have further solved the key problems of low stem cell differentiation efficiency and poor *in vivo* survival. These advances have formed a complete translational research chain from “cell preparation” to “*in vivo* colonization”, providing a solid foundation for the clinical translation of IDD stem cell therapy.

### 3.3 Cell transplantation techniques and their biological effects

Cell transplantation aims to restore NPCularity and ECM synthesis. Two main delivery methods have advanced. Direct cell suspension injection and cell carrier-based systems are used. Direct injection is simple and minimally invasive. But cell retention and survival are low. The degenerated disc’s harsh environment is to blame (Dai et al., 2025).

Biomaterial-based carriers address these challenges. Hydrogels and porous scaffolds encapsulate cells. They mimic the native ECM and improve cell viability (Wang Y. et al., 2021; Zhang X. et al., 2025). Gelatin methacryloyl (GelMA), hyaluronic acid-based hydrogels, and decellularized NP hydrogels are common. They are biocompatible and match the NP’s mechanical properties. This supports cell adhesion and proliferation (Xu et al., 2021; Dai et al., 2025).

GelMA-based hydrogels loaded with NPCs enhance proliferation. Hydrogel stiffness influences cell behavior and matrix deposition (Xu et al., 2021). Composite hydrogel microspheres with Prussian blue nanozymes protect MSCs. This hydrogel microsphere reduces ROS-induced damage and promotes intervertebral disc regeneration (Zhang X. et al., 2025). These biomimetic scaffolds are key to improving transplantation outcomes.

Cell survival, proliferation, and ECM synthesis determine therapy success. Preconditioning NPSCs improves their survival. Quiescent NPSCs have decreased apoptosis. They adapt metabolically to harsh conditions (Chen Q. et al., 2023). This preserves disc height and structure post-transplantation.

Pharmacological pretreatment also helps. Agents like bardoxolone methyl and myricetin protect transplanted NPCs. They reduce oxidative stress and apoptosis. They activate antioxidant pathways like Nrf2 and SIRT1/PGC-1α (Tian et al., 2021; Xie et al., 2023). These approaches boost the biological performance of transplanted cells.

Cell carriers can also release bioactive factors. Porous leaf-stack structured particles load BMSCs and TGF-β3. They release growth factors sustainably. This promotes chondrogenic differentiation and IDD regeneration (Kim et al., 2020). Hydrogel systems co-deliver MSCs and Salvianolic acid B. They activate JAK2-STAT3 signaling. This reduces apoptosis and delays disc degeneration (Hu et al., 2022). These composite systems integrate cells, biomaterials, and drugs.

Immunological factors are important for transplantation. Donor-recipient sex mismatch affects graft survival. When male donor neural progenitor cells are transplanted into a female host for spinal cord injury, T-cell infiltration and excessive vascularization can occur (Pitonak et al., 2022). The disc is somewhat immune-privileged. But allogeneic or xenogeneic cells can trigger immune responses. Modulating immunity with immunomodulatory agents is crucial (Skartsis et al., 2023; Wang et al., 2023b). This enhances graft survival and integration.

**FIGURE 2:**
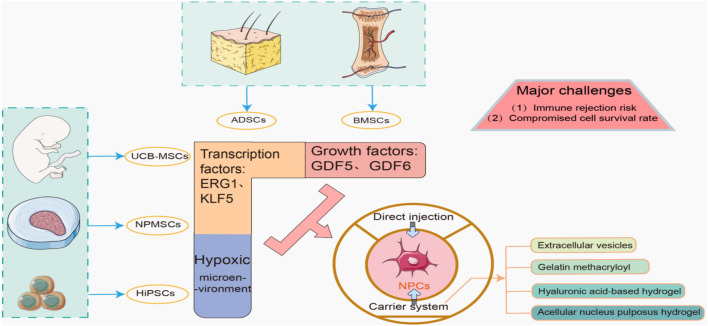
Scheme of Diverse Stem Cell Sources: Nucleus Pulposus Cell Differentiation Regulation and Delivery Strategies in Cell Therapy for Intervertebral Disc Degeneration.

In summary, biomaterial-based delivery systems improve cell transplantation. They protect cells, enhance survival, and promote ECM synthesis. Preconditioning and bioactive factors further optimize outcomes. Addressing immunological compatibility is essential. (See Figure 2 for details). Future research will refine these strategies for clinical use.

### 3.4 Challenges faced by cell therapy

The survival of transplanted cells in the harsh intervertebral disc microenvironment is the primary problem. As described in Section 6.3, the degenerative intervertebral disc microenvironment is a common obstacle for all biomedical engineering therapies. Mesenchymal stem cells (MSCs) often undergo rapid apoptosis or senescence in this environment, thereby limiting their regenerative potential. To address this core pain point, strategies to enhance cell stress resistance are currently being explored. TGF-β3 pretreatment can reduce the adverse effects of low pH on cell viability and matrix accumulation (Gansau and Buckley, 2021). Biomaterial carriers such as hydrogels and microscaffolds provide support, improve cell survival and retention rates, and prevent cell leakage under high intervertebral disc pressure. In recent years, researchers have developed a variety of intervention strategies that can effectively improve cell microenvironment tolerance, and some of these strategies have shown more stable effects on long-term *in vivo* cell survival rates: for example, a delivery system using leaf stack structure (LSS) porous particles loaded with human bone marrow mesenchymal stem cells (hBMSCs) and transforming growth factor-β3 (TGF-β3) can achieve sustained release of TGF-β3 (up to 18 days) and maintain stable cell adhesion. This strategy has been verified in a beagle (large animal) model, and can provide a suitable environment for hBMSCs to differentiate into chondrocytes and effectively induce intervertebral disc regeneration (Kim et al., 2020) in addition, encapsulating salvianolic acid B (SalB) with bone marrow mesenchymal stem cells in 1% HAMA hydrogel can significantly reduce the cell apoptosis rate by activating the JAK2-STAT3 pathway, as verified in in vitro experiments (Hu et al., 2022); in addition, strategies to improve cell antioxidant capacity through pretreatment have also shown stable effects, such as pretreatment of nucleus pulposus cells with bardoxolone methyl (BARD) can inhibit compression-induced oxidative stress and apoptosis by activating the Nrf2 signaling pathway (Tian et al., 2021) and myricetin pretreatment can protect nucleus pulposus-derived mesenchymal stem cells from oxidative damage and delay senescence by regulating the SIRT1/PGC-1α pathway (Xie et al., 2023). These strategies maintain a high cell survival rate *in vivo* for a long time through biomaterial carrier protection, drug pretreatment, or gene regulation.

Among the above-mentioned various cell protection strategies, biomaterial delivery carriers have become one of the most widely used technical solutions due to their multiple advantages of mechanical support, cell anchoring, and microenvironment regulation. Biomaterial carriers such as hydrogels and microsphere scaffolds can provide support for cells, improve cell survival and retention rates, and prevent cell leakage under high intervertebral disc pressure. For example, esterase-responsive kartogenin composite hydrogel microspheres (GHKM) can simulate the nucleus pulposus extracellular matrix, provide adhesion and mechanical support for cells, and help cells adapt to adverse microenvironments through responsive release of kartogenin, maintaining the survival of nucleus pulposus cells and promoting extracellular matrix synthesis in an 8-week experiment in a rat model (Dai et al., 2025).

On the basis of solving the cell survival problem, the regulation of directed differentiation of transplanted cells is another challenge. The intervertebral disc contains specialized cell types, and nucleus pulposus progenitor cells can differentiate into nucleus pulposus-like cells for intervertebral disc repair (Wang P. et al., 2025). However, inflammatory factors and oxidative stress in the degenerative intervertebral disc microenvironment are the main pathological factors related to intervertebral disc degeneration (Ząbek et al., 2025).

Advanced biomedical engineering means provide solutions. CRISPR-mediated gene modification targets regulatory factors such as ZNF865 to enhance matrix deposition in engineered intervertebral disc models (Levis et al., 2025). HAV peptide-functionalized GelMA microspheres promote cell-cell interactions, enhance extracellular matrix secretion by nucleus pulposus cells and promote nucleus pulposus tissue regeneration (Tian et al., 2025).

In addition to the two major technical problems of cell survival and directed differentiation, immune rejection and ethical issues constitute additional obstacles.

Although the intervertebral disc has immune privilege characteristics, it is not completely immunologically isolated, and allogeneic or xenogeneic cells may still trigger immune reactions, leading to graft rejection and inflammation. Autologous cells can avoid immunogenicity problems, but there are problems such as donor site injury, limited cell sources, and decreased regenerative ability with age.

From the ethical and safety perspectives, the clinical application of some stem cell types still has many controversies. For example, embryonic stem cells and human induced pluripotent stem cells (hiPSCs) have the risk of tumorigenicity and uncontrolled differentiation. Human urine-derived stem cell exosomes can improve intervertebral disc degeneration through related mechanisms (Guo et al., 2021) but the long-term safety and effectiveness of cell-free therapies still need comprehensive evaluation.

Based on the above research progress and existing challenges, cell therapy for IDD still faces significant challenges, and cell survival, directed differentiation, immune reactions, and ethical issues are core problems. Integrating biomaterials, gene editing, and cell-free therapies provides directions for solving these problems, and interdisciplinary research and clinical trials will promote the translation of these strategies to clinical practice.

## 4 Tissue engineering scaffold materials and their applications in intervertebral disc repair

### 4.1 Types and characteristics of scaffold materials

Scaffold materials are critical for IDD tissue engineering. They must support cell activities and match native disc mechanics. Two main categories are widely used: natural biomaterials and synthetic polymers.

Natural materials like collagen, chitosan, and silk fibroin are popular. They are highly biocompatible. They mimic the disc’s native ECM (Friedmann et al., 2021; Lin et al., 2023; Tang et al., 2024). Collagen is the main structural protein in the disc. Injectable collagen hydrogels loaded with ADSCs stabilize disc height. They reduce degenerative features in sheep models (Friedmann et al., 2021). But complete disc height restoration remains difficult.

Chitosan scaffolds with high deacetylation have interconnected pores. This favors stem cell adhesion and prolonged NP-like differentiation (Tang et al., 2024). They upregulate ECM-related gene expression. This makes them suitable for direct implantation. Silk fibroin (SF) has excellent physical and chemical properties. It is biocompatible and mechanically strong. SF-based biomaterials support disc tissue engineering. They show promise for clinical translation (Lin et al., 2023).

Synthetic polymers offer tunable properties. Poly (lactic-co-glycolic acid) (PLGA) and polycaprolactone (PCL) are common. They have controllable degradation rates. They are easy to fabricate, especially with additive manufacturing (Angili et al., 2023). PLGA composite scaffolds support bone regeneration and spinal fusion. Nanoparticle incorporation enhances osteogenic potential (Li Y. et al., 2025).

PCL has excellent mechanical integrity and stability. It mimics native tissue structural features. PLA-based scaffolds coated with alginate and magnesium oxide nanoparticles improve antibacterial activity. Increasing MgO content can increase the compressive strength and elastic modulus of the scaffold, and studies have been conducted on its biological properties including biocompatibility (Angili et al., 2023). 3D printing allows precise control over scaffold geometry. This enables tailored implant properties (Yoshida et al., 2022).

Biocompatibility is critical for scaffold integration. Natural and synthetic materials differ in physical and biological properties. These affect cell-scaffold interactions (Wang F. et al., 2023). Composite scaffolds combine synthetic polymers with natural ECM components. This balances mechanical strength and cell-favorable environments. Degradation products must be non-toxic. They should not induce inflammation.

Electrospun nanofiber scaffolds mimic the AF’s fibrous structure. They have aligned nanofibers that guide cell orientation. Both natural and synthetic polymers are used. Reinforcing hydrogels with electrospun nanofibers improves disc repair. Electrospun nanofiber-reinforced hydrogels can improve the mechanical properties and biocompatibility of nucleus pulposus scaffolds (Li C. et al., 2022). Piezoelectric scaffolds convert mechanical loading into electrical stimulation. They modulate cell behavior and immune responses (Huang et al., 2024) This offers new therapeutic possibilities.

In summary, scaffold material choice depends on multiple factors. Biocompatibility, mechanical strength, degradation profile, and cell support are key. Combining natural and synthetic materials, advanced fabrication, and bioactive agents is promising. These approaches develop clinically translatable scaffolds for disc repair (See Table 1 for details).

**TABLE 1:** Performance comparison of scaffold materials for intervertebral disc degeneration repair.

Scaffold category and representative material	Core advantages	Key performance summary (integration of biocompatibility, mechanical strength, degradation rate)	Clinical application and translational potential	Core limitations	References
Natural BiomaterialsCollagen	1. Core structural protein of the intervertebral disc (IDD) extracellular matrix (ECM), which perfectly mimics the native microenvironment 2. Aligned structure guides oriented cell arrangement and restores the mechanical stability of the annulus fibrosus (AF) 3. Serves as a carrier for cells/active factors to improve the survival rate of transplanted cells	Excellent biocompatibility with no immunogenicity; the tensile strength of aligned hydrogel can reach ∼5 MPa, matching the mechanical properties of native AF; degradation rate can be precisely regulated by crosslinking agents to match the rate of tissue regeneration	★★★★★ Has entered early clinical exploration, and is one of the natural materials with the fastest clinical translation progress. It is suitable for AF tear repair and minimally invasive nucleus pulposus (NP) filling, and can be combined with mesenchymal stem cells (MSCs)/growth factors to repair early-to-moderate intervertebral disc degeneration (IDD)	Poor mechanical properties and rapid degradation in the uncrosslinked state; batch-to-batch variation in animal-derived materials; limited injectability at high concentrations	Ma et al. (2024a), Montesinos et al. (2023), Qiao et al. (2025)
Natural BiomaterialsSilk Fibroin (SF)	1. Enables biomimetic construction of AF-NP integrated scaffolds to accurately simulate the native lamellar structure of AF 2. Microchannel structure promotes cell infiltration and nutrient delivery, restoring IDD height and dynamic mechanical properties 3. Achieves 68% IDD height retention rate in rabbit degeneration models, with long-term inflammation inhibition	Excellent biocompatibility, low immunogenicity and no cytotoxicity; mechanical properties can be regulated in a wide range through preparation processes to match the physiological mechanics of each region of the IDD; *in vivo* degradation cycle is 6–12 months, highly matching the regeneration cycle	★★★★☆ Suitable for total disc replacement in moderate-to-severe IDD and repair of large AF defects. Long-term mechanical stability has been verified in large animal models, with large-scale production and translation potential	Complex processing technology; batch-to-batch performance variation of natural materials; long-term safety of degradation products needs clinical verification	Madhu et al. (2024), Mirzaeipoueinak et al. (2023), Özkale et al. (2021)
Natural BiomaterialsChitosan	1. Interconnected pore structure supports stem cell adhesion and NP-like differentiation 2. Can be constructed into injectable composite hydrogels, achieving 12 months of stable 1DD height in large animal models 3. Possesses natural antibacterial activity to reduce the risk of postoperative infection	Good biocompatibility with no obvious inflammatory reaction after *in vivo* implantation; moderate mechanical properties, which can be enhanced by crosslinking/compositing nanomaterials to match the compression modulus of native NP; degradation rate can be precisely regulated by crosslinking degree	★★★★☆ Suitable for minimally invasive injection therapy of moderate IDD (Pfirrmann grade III-IV). It can be combined with MSCs/growth factors/anti-inflammatory drugs to achieve multi-effect repair, and long-term safety has been verified in large animal models with outstanding translational potential	Insufficient mechanical matching with native IDD under high load; poor cell adhesion of pure material, requiring composite modification	Ma et al. (2024b), Nakielski et al. (2023), Ongini et al. (2025), Ren et al. (2025b)
Natural BiomaterialsHyaluronic Acid (HA)/Chondroitin Sulfate	1. Core component of NP ECM, with excellent water retention and viscoelasticity, accurately simulating the physiological rheological properties of native NP 2. Can be constructed into injectable *in-situ* crosslinking hydrogels, suitable for minimally invasive puncture administration 3. Serves as a delivery carrier for growth factors to achieve long-term sustained release	Excellent biocompatibility, naturally present in IDD tissue with no immunogenicity; low-to-moderate mechanical properties of pure material, which can be improved by dual-network crosslinking/composite modification; pure HA degrades rapidly, and the cycle can be extended to 3–6 months after crosslinking	★★★★★ Approved in Europe and the United States for viscoelastic supplementation therapy of IDD, it is currently the most widely used scaffold material for clinical IDD repair, and is suitable for minimally invasive injection therapy of early-to-moderate IDD	Insufficient mechanical strength and rapid degradation of pure material; unable to repair AF structural defects, only applicable to early degeneration	Munda and Velnar (2024), Niemeyer et al. (2024), Penolazzi et al. (2022), Ren et al. (2025b)
Natural BiomaterialsDecellularized Nucleus Pulposus Matrix (DNPM)/Intervertebral Disc Matrix	1. Fully retains the biochemical components and microstructure of IDD tissue, providing tissue-specific differentiation signals 2. Can be constructed into injectable hydrogels for minimally invasive scenarios 3. Can guide endogenous stem cell homing without exogenous cell transplantation	Good biocompatibility, low immunogenicity after decellularization; low-to-moderate mechanical properties, which can be improved by crosslinking/compositing polymer materials; degradation rate matches the rate of tissue regeneration	★★★☆☆ Suitable for cell/drug delivery and tissue regeneration in early-to-moderate IDD. Its tissue-specific advantage gives it unique value in the field of precise regeneration, and its effectiveness has only been verified in small animal models at present	Limited source of human tissue with significant batch-to-batch differences; decellularization process may damage matrix activity; insufficient mechanical properties	Jiang et al. (2024), Nakielski et al. (2023)
Synthetic Polymers Polycaprolactone (PCL)	1. Can be constructed into aligned nanofiber structure to accurately simulate the native angle-ply microstructure of AF, guiding oriented cell arrangement and matrix deposition 2.3D printing can prepare flexible porous scaffolds with widely adjustable mechanical properties and long-term mechanical stability 3. Slow degradation provides long-term structural support	Good biocompatibility with no obvious cytotoxicity, and cell affinity can be improved by surface modification; excellent mechanical properties, the tensile properties of aligned nanofiber scaffolds can fully match human native AF; *in vivo* complete degradation cycle is 2–4 years, suitable for long-term mechanical support	★★★★★ Suitable for total disc replacement in severe IDD (Pfirrmann grade V) and repair of large AF defects. It is the core base material for tissue-engineered total IDD scaffolds at present, with long-term mechanical stability verified in large animal models and outstanding translational potential	Poor cell adhesion caused by intrinsic hydrophobicity; excessively long degradation cycle with risk of chronic foreign body reaction; lack of bioactivity	Marshall et al. (2021), Özkale et al. (2021), Öztürk et al. (2024)
Synthetic PolymersPoly (lactic-co-glycolic acid) (PLGA)	1. Degradation rate and mechanical properties can be precisely regulated by monomer ratio, which is the gold standard material for microsphere delivery of growth factors/gene drugs 2. Can achieve sustained release of growth factors for several weeks, promoting the differentiation of stem cells into NP-like cells 3. Mature industrial production process	Good biocompatibility, FDA-approved clinical implant material; mechanical properties can be regulated in a wide range through process optimization; degradation cycle is adjustable from 1 month to 1 year, with acidic degradation products	★★★★☆ Mainly used as a delivery carrier for growth factors/gene drugs/stem cells, suitable for minimally invasive injection therapy of early-to-moderate IDD. Its effectiveness has been verified in multiple preclinical studies, with rapid clinical translation conditions	Acidic degradation products tend to induce local aseptic inflammation; poor cell affinity; unable to provide long-term mechanical support	Jiang et al. (2024), Li et al. (2024c), Maggio et al. (2020), Wang et al. (2021b)
Synthetic PolymersPolylactic Acid (PLA)/Flexible Polylactic Acid (FPLA)	1. High 3D printing molding accuracy, can prepare personalized porous scaffolds to adapt to patient-specific anatomical structures 2. The viscoelasticity of FPLA can accurately match the physiological mechanics of native IDD, with better mechanical stability than standard PLA 3. Can be composited and modified to endow antibacterial activity	Good biocompatibility, FDA-approved clinical implant material; good mechanical properties, which can be regulated in a wide range through structural design/material composite; *in vivo* complete degradation cycle is 1–2 years, with slower degradation and longer stability of FPLA	★★★☆☆ Suitable for the preparation of personalized IDD scaffolds and spinal fusion cages, with translational value in the field of customized IDD repair	High intrinsic brittleness and poor fatigue resistance; acidic degradation products tend to induce local inflammation; lack of bioactivity	Marshall et al. (2021), Öztürk et al. (2024)
Advanced Composite ScaffoldsElectrospun Nanofibrous Scaffolds	1. Can be prepared into core-shell structure to achieve differential sequential release of anti-inflammatory drugs and pro-regenerative factors 2. Can accurately construct the angle-ply structure consistent with native AF, promoting ordered tissue regeneration 3. High specific surface area improves the efficiency of cell adhesion and matrix deposition	Good biocompatibility, supporting long-term cell survival and oriented differentiation with no obvious inflammatory reaction *in vivo*; the tensile and fatigue properties of aligned scaffolds can match native AF; degradation rate can be precisely regulated by substrate selection	★★★★★ It is the gold standard scaffold type for AF defect repair at present, suitable for AF repair after discectomy and construction of total IDD tissue engineering. Long-term effectiveness has been verified in large animal models, with outstanding clinical translation prospects	Limited nutrient diffusion in thick scaffolds; complex large-scale production process with high difficulty in quality control	Naha and Driscoll (2024), Neidlinger-Wilke et al. (2024), Özkale et al. (2021)
Advanced Composite ScaffoldsPiezoelectric Scaffolds	1. Can simulate the piezoelectric effect of native IDD, convert physiological spinal load into endogenous electrical signals, regulate cell anabolism and anti-inflammatory phenotype without exogenous power supply 2. Can be composited to construct injectable/implantable multifunctional scaffolds to realize mechanical-biological signal conversion	Good biocompatibility, commonly used substrates (PVDF, PLA) have excellent biosafety; good mechanical properties, matching the physiological compression modulus and fatigue resistance of IDD; degradation rate can be regulated by substrate selection	★★★☆☆ Suitable for IDD repair related to mechanical load, providing a new direction for non-pharmaceutical/non-cellular therapy of IDD with outstanding long-term therapeutic potential. Its effectiveness has only been verified in small animal models at present	Long-term *in vivo* piezoelectric stability and safety have not been fully verified in large animal models; high difficulty in signal regulation	Hao et al. (2026)
Advanced Composite Scaffolds3D-Printed Integrated Composite Scaffolds	1. Multi-material printing can one-time construct an integrated structure of outer aligned AF + inner porous NP, accurately replicating the anatomical structure and mechanical anisotropy of native IDD 2. Can realize spatial gradient regulation of porosity and mechanical properties, integrating drug/cell delivery functions 3. Supports personalized customization	Good biocompatibility, multi-material composite balances structural support and cell activity, supporting oriented differentiation of stem cells; excellent mechanical properties, can accurately match the dynamic and static mechanics of native IDD with outstanding fatigue resistance; degradation rate can be gradient-regulated to adapt to the regeneration needs of different regions	★★★★★ Suitable for total disc replacement in severe IDD, it is the core development direction of tissue-engineered IDD in the future, supports personalized customization, and has shown excellent mechanical stability and tissue regeneration effect in large animal models	High threshold of multi-material printing equipment and process; high difficulty in minimally invasive implantation; matching between degradation and regeneration still needs optimization	Matyas et al. (2021), Öztürk et al. (2024)
Functionalized/Smart ScaffoldsPeptide-Functionalized Hydrogels	1. Functional peptide modification can significantly enhance cell adhesion, survival and phenotype maintenance 2. Can improve cell retention rate in degeneration models, and ameliorate IDD height and matrix synthesis 3. Can achieve cell-specific targeting to improve the accuracy of gene/drug delivery	Excellent biocompatibility, functional peptides are derived from natural proteins with no immunogenicity; moderate mechanical properties, which can be regulated by crosslinking degree to match the viscoelasticity of NP; degradation rate can be regulated by matrix material and crosslinking method	★★★☆☆ Suitable for cell/drug delivery therapy of early-to-moderate IDD, can significantly improve the therapeutic effect of transplanted cells, and its effectiveness has been verified in multiple preclinical studies	Peptides are easily enzymolyzed *in vivo* with poor stability; high synthesis cost and great difficulty in large-scale production	Lu and Lin (2021), Munda and Velnar (2024)
Functionalized/Smart ScaffoldsGraphene Oxide (GO) Hybrid Hydrogels	1. GO can strongly bind growth factors, achieve long-term sustained release for several weeks while maintaining high bioactivity, and promote matrix synthesis 2. Can improve the mechanical properties of hydrogels by 2–5 times, and endow near-infrared responsive drug controlled release ability 3. Possesses excellent free radical scavenging ability to alleviate oxidative stress	Good biocompatibility at low concentration with no obvious cytotoxicity; significantly improved mechanical properties, with greatly enhanced compression modulus and tensile strength; the degradation of hydrogel matrix is controllable, while the *in vivo* metabolic pathway and long-term safety of GO remain to be verified	★★★☆☆ Suitable for IDD repair requiring long-term controlled release of growth factors, can simultaneously achieve mechanical enhancement, anti-oxidation and drug controlled release, and has shown excellent regeneration effect in preclinical studies	The long-term *in vivo* biosafety and metabolic pathway of GO have not been clarified; there is a risk of cytotoxicity at high concentration	Ongini et al. (2025), Song et al. (2023)
Functionalized/Smart ScaffoldsMicroenvironment-Responsive Smart Hydrogels	1. Can respond to the pathological characteristics of degenerated IDD such as high MMP, high ROS and acidic pH, realize precise on-demand release of drugs/genes, and reduce off-target effects 2. Can achieve adaptive matching between scaffold degradation and tissue regeneration 3. Can encapsulate multiple therapeutic components to realize sequential combination therapy	Excellent biocompatibility, responsive functional groups have no obvious toxicity, and can accurately adapt to pathophysiological changes; moderate mechanical properties, which can be regulated by crosslinking system to match the viscoelasticity of NP; degradation/drug release rate is positively correlated with local inflammation degree, with adaptive regulation	★★★★☆ Suitable for inflammation-mediated progressive IDD, can achieve precise targeted therapy and reduce side effects. It is the core development direction of targeted drug delivery for IDD in the future, and excellent therapeutic effects have been verified in multiple animal models	High difficulty in balancing response sensitivity and specificity; long-term *in vivo* response stability needs to be verified in large animals; high industrialization difficulty	Tang et al. (2024), Xiao et al. (2020)
Functionalized/Smart ScaffoldsGene Delivery Functionalized Scaffolds	1. Can achieve long-term local delivery of nucleic acid drugs, protect nucleic acids from enzymatic degradation, reduce off-target effects, and achieve stable gene regulation for up to 28 days 2. Can be combined with stem cell therapy to enhance its therapeutic capacity through gene editing 3. Can reverse the core mechanism of degeneration at the molecular level	Non-viral vector composite scaffolds have good biocompatibility and low immunogenicity; viral vectors require strict evaluation of immune safety; moderate mechanical properties, which can be regulated by hydrogel matrix; degradation rate matches the gene release cycle and tissue regeneration rate	★★★☆☆ Suitable for gene-cell combination therapy of refractory and progressive IDD, it is the cutting-edge direction of IDD regenerative medicine, and preclinical studies have shown radical therapeutic potential	Viral vectors have risks of immunogenicity and insertional mutation; non-viral vectors have low transfection efficiency; long-term safety and ethical issues need to be verified	Sun et al. (2024), Velnar and Gradisnik (2023), Wang et al. (2023c), Xiao et al. (2020)
Multifunctional Delivery ScaffoldsAligned Core-Shell Nanofibrous Scaffolds	1. Core-shell structure realizes regional encapsulation and sequential release of anti-inflammatory drugs and pro-regenerative factors, with early anti-inflammation and late pro-regeneration effects 2. Aligned structure guides oriented arrangement of AF cells and promotes ordered tissue regeneration 3. Can achieve sustained release of growth factors for up to 1 month	Good biocompatibility, no obvious cytotoxicity and inflammatory reaction, supporting long-term cell survival and oriented differentiation; good mechanical properties, the tensile properties of aligned fibers can match native AF; degradation and drug release cycle can be precisely regulated by core-shell materials	★★★★☆ Suitable for AF defect repair after discectomy, can simultaneously solve the two major clinical pain points of postoperative inflammation and AF healing, significantly reduce the recurrence rate, and has extremely high clinical translation value	Complex preparation process with high difficulty in industrial quality control; limited nutrient diffusion in thick scaffolds	Naha and Driscoll (2024), Neidlinger-Wilke et al. (2024)
Multifunctional Delivery ScaffoldsHydrogel Microsphere Delivery Scaffolds	1. Excellent injectability, can achieve intradiscal delivery through minimally invasive puncture to avoid open surgery 2. Can precisely regulate stem cell behavior through the physicochemical properties of microspheres, and soft matrix can guide stem cells to differentiate into NP-like cells 3. Can realize co-encapsulation and long-term sustained release of multiple components	Excellent biocompatibility, all are clinically commonly used degradable materials with no immunogenicity, supporting three-dimensional growth of stem cells; mechanical properties can be regulated in a wide range to match the mechanical microenvironment of different regions of IDD; degradation cycle is adjustable from weeks to months, matching the regeneration rate	★★★★★ It is the core carrier type of minimally invasive IDD regenerative therapy at present, suitable for minimally invasive injection therapy of IDD at all stages, can be used alone or in combination with cells/drugs/genes. Long-term safety and effectiveness have been verified in large animal models, with extremely fast clinical translation progress	High difficulty in controlling the uniformity of microsphere particle size; risk of displacement after injection; unable to repair large AF defects	Levis et al. (2023), Li et al. (2022a), Wang et al. (2021b), Yadav et al. (2025), Zhao et al. (2024)

### 4.2 Scaffold structure design and cell interaction

Scaffold structure design directly impacts disc tissue engineering success. It influences cell adhesion, migration, and differentiation. Porosity and microtopology are crucial parameters.

Biomimetic scaffolds with interconnected pores facilitate nutrient diffusion. They remove waste and create a favorable microenvironment. A novel silk fibroin scaffold (BMI-SF) has oriented cross-microchannels in the AF. It has interconnected pores in the NP region (Zhang T. et al., 2025). This scaffold is biocompatible and biodegradable. Microchannels guide cell infiltration and neovascularization. *In vivo*, it restores IDD height and mechanical properties. This shows scaffold microstructure promotes functional regeneration.

Microtopological cues affect cellular behavior at the molecular level. Scaffolds with aligned collagen fibrils promote AF cell and MSC alignment. Primary AF cells and RBMSCs cultured on Col-I HA hydrogel scaffolds show increased levels of CD146 and Acta2, indicating a transition to a contractile phenotype (Yadav et al., 2025). A hyaluronan-coated type-I collagen hydrogel has aligned fibrils. It has high tensile strength matching native AF tissue. It supports cell viability and alignment. *In vivo*, it restores mechanics and deposits collagen and glycosaminoglycans (Yadav et al., 2025). But fibrotic changes were observed, indicating complex repair remodeling.

Scaffold surface functionalization enhances cell attachment. Bioactive peptides like RGD sequences promote tissue formation. Peptide-functionalized hydrogels deliver cells for IDD regeneration. A polymer-peptide hydrogel with laminin-mimetic peptides (IKVAV and AG73) carries NPCs (Barcellona et al., 2021). It promotes viability and biosynthetic activity *in vitro*. *In vivo*, it supports NP-specific protein expression and disc height. These peptide motifs enhance cell retention and phenotypic expression.

Multifunctional nanofibrous scaffolds co-deliver therapeutic agents. Electrospun core-shell scaffolds load TGF-β3 and ibuprofen. They have rapid anti-inflammatory effects. They sustain ECM formation (Han et al., 2022). Their angle-ply microstructure mimics native AF. This enhances ECM deposition and maintains mechanical properties. Modulating inflammation while providing structural cues improves regeneration.

In summary, scaffold structure design with porous architecture and microtopology is critical. Peptide functionalization promotes cell-scaffold interactions. Combining structural and biochemical cues creates a conducive environment. Future designs may integrate spatially controlled microstructures and bioactive modifications. This optimizes cell behavior and disc repair.

### 4.3 Combined application of scaffolds and growth factors

Integrating scaffolds with growth factors is a cutting-edge IDD strategy. Growth factors like TGF-β1 and BMPs promote tissue repair. Scaffolds act as 3D frameworks and delivery vehicles. They enable controlled, localized release of growth factors. This maintains bioactivity over time.

A decellularized NP matrix/chitosan hydrogel loaded with TGF-β3 is effective. It enhances NPSC proliferation. It upregulates collagen-I, collagen-II, and aggrecan genes (Kuang et al., 2021). These are key ECM components. Controlled release overcomes rapid diffusion and degradation issues.

Biomimetic scaffolds replicate the disc’s hierarchical and biochemical environment. This boosts growth factor delivery efficacy. Micro/nanofibrous scaffolds encapsulate bFGF. They are made via hyaluronan micro-sol electrospinning and collagen self-assembly (Tu et al., 2023). Sustained bFGF release promotes AF cell adhesion and proliferation. It simulates native ECM microstructure. This facilitates endogenous regeneration after AF injury.

Advanced hybrid materials optimize growth factor delivery. Graphene oxide (GO)-self-assembling peptide hydrogels deliver TGF-β3 (Ligorio et al., 2021). GO flakes bind TGF-β3 strongly. This enables slow, sustained release while preserving bioactivity. Bovine NPCs cultured in this scaffold upregulate NP-specific genes. They deposit an NP-like ECM rich in aggrecan and collagen II. Nanomaterials like GO enhance mechanical properties and cellular interactions.

Multifunctional scaffolds co-deliver multiple bioactive agents. Electrospun core-shell nanofibrous scaffolds load TGF-β3 and ibuprofen (Han et al., 2022). TGF-β3 sustains ECM synthesis. Ibuprofen improves the inflammatory milieu. Together, they support AF repair and maintain mechanical integrity. This dual-delivery system addresses the multifaceted pathology of IDD.

*In vivo* studies validate scaffold-growth factor combinations. Peptide-functionalized double network hydrogels incorporate GDF-5 (Ho et al., 2023). They mimic native IDD structure. They recruit endogenous MSCs and facilitate differentiation. This reconstructs injured discs with native-like mechanical properties. These findings show growth factor-loaded scaffolds actively modulate cell behavior.

In summary, combining scaffolds and growth factors enables controlled release. It enhances ECM synthesis and functional tissue regeneration. Scaffold material design and growth factor incorporation methods are critical. Nanomaterials and multifunctional delivery systems further improve outcomes. Future research will optimize these parameters for clinical translation.

However, the *in vivo* functional reconstruction achieved by these growth factor-loaded scaffolds is highly dependent on their adaptability to the physiological mechanical microenvironment of the intervertebral disc. This critical interplay between biochemical regulatory cues and the native mechanical biophysical microenvironment of the intervertebral disc will be systematically examined in the following section.

### 4.4 Current status of clinical translation of scaffold materials

Scaffold materials have shown promise in animal models for IDD repair. Hydrogels, electrospun fibers, and 3D-printed constructs have been tested. Injectable chitosan hydrogels stabilize disc height in sheep. They mitigate degeneration for up to 12 months (Schmitt et al., 2021). Electrospun nanofibrous scaffolds replicate the AF’s lamellar structure. They support cell adhesion and match native tissue mechanics (Shamsah et al., 2019). 3D-printed PLA scaffolds have viscoelastic properties matching IDD motion segments. They promote MSC fibrocartilaginous differentiation (Marshall et al., 2021). These preclinical studies show scaffolds provide structural support and a conducive microenvironment.

But clinical translation faces significant challenges. No FDA-approved intradiscal therapies exist for discogenic back pain (Ju et al., 2020). Achieving sufficient mechanical robustness is a bottleneck. Scaffolds must integrate with the degenerated disc’s harsh environment (Zhao et al., 2025). Fibrin-genipin hydrogels bond AF lesions in rats. Genipin-crosslinked hydrogels have potential for clinical translation in the treatment of annulus fibrosus healing in rats (Wang Z. et al., 2023). Scaffold migration, degradation rates, and immune responses are concerns. The AF’s complex angle-ply structure complicates scaffold design (Zhou D. et al., 2024). Scaling production and addressing immunogenicity are also hurdles (Wang X. et al., 2025).

Pilot clinical studies explore novel scaffold-based AF repair. A non-woven PET scaffold integrated into goat cervical spine tissue. It prevented reherniation and supported integration (Ongini et al., 2025). No migration or degeneration was seen over 4 weeks. Translating this to humans requires addressing scale and biomechanical differences. Multifunctional scaffolds co-delivering anti-inflammatory agents and growth factors show promise. But they add regulatory and manufacturing complexity.

In summary, scaffold materials have therapeutic potential in preclinical models. Mechanical, biological, and regulatory challenges hinder clinical translation. Refining scaffold design to match native tissue mechanics is necessary. Large-animal studies and well-designed clinical trials are essential. 3D bioprinting and advanced functionalization may overcome current bottlenecks. This will facilitate the adoption of scaffold-based therapies for IDD. (See Table 1 for details).

## 5 Gene therapy and molecular regulation strategies

### 5.1 Target selection for gene therapy

Gene therapy for IDD aims to balance anabolic and catabolic activities. It modulates genes involved in ECM synthesis and degradation. Promoting matrix synthesis genes is a key focus. Collagen type II (COL2A1) and aggrecan are targeted. Inhibiting matrix-degrading enzymes like MMPs is also important.

IDD is marked by reduced ECM synthesis and increased degradation. This undermines disc integrity (Ząbek et al., 2025). SOX9, a transcription factor for chondrogenesis, is critical. Its conditional deletion accelerates degeneration via the β-catenin/CCL2 pathway (Aboushaala et al., 2025). Upregulating SOX9 or its targets may counteract matrix breakdown.

Catabolic enzymes like MMP9 and MMP13 are linked to IDD. They are potential molecular targets (Francisco et al., 2023). RNA interference (RNAi) silences genes involved in degeneration (Takeoka et al., 2020). Autophagy-related genes like EP300 and SIRT5 maintain cellular homeostasis. Activation of the EP300-SIRT5 signaling axis inhibits NPC senescence by promoting autophagy (Liu et al., 2025). Enhancing autophagy via EP300-SIRT5 signaling preserves ECM integrity.

Inflammation and apoptosis-related genes are also targeted. Inflammation is one of the pathophysiological processes of intervertebral disc degeneration (IDD), and IL-1β and TNF-α play both promoting and potential alleviating dual roles in IDD (Ren Q. et al., 2025). Gene therapy suppresses these cytokines or their signaling pathways. Adrenomedullin (ADM) inhibits MAPK signaling. This reduces NPC senescence and apoptosis (Ren L. et al., 2025). Targeting the STING pathway with siRNA inhibits pyroptosis. This delays IDD progression (Zhou et al., 2026).

Oxidative stress and cellular survival genes are emerging targets. MSCs may alleviate IDD by modulating oxidative stress-related genes (Zhao et al., 2022). Genes like p53 regulate senescence and apoptosis. p53 plays an important role in maintaining intervertebral disc microenvironment homeostasis (Wang Y. et al., 2023). Lactylation-related genes like LRP1 are novel targets (Yang Y. et al., 2025).

**FIGURE 3:**
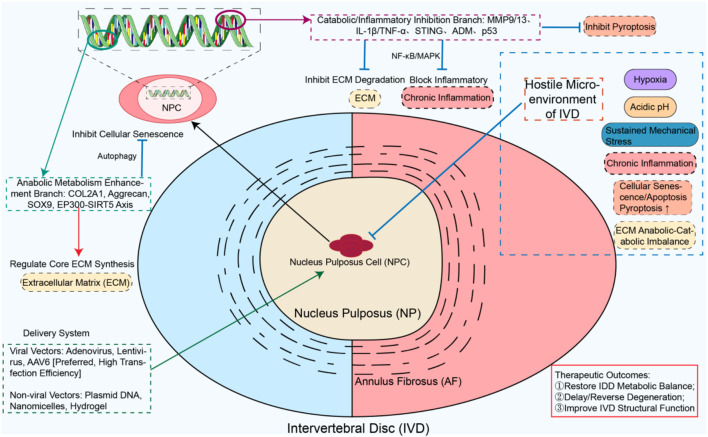
Gene Therapy Restores Disc Homeostasis via Bidirectional Regulation In the hostile IVD milieu (hypoxia, acidity, mechanical stress), viral/non-viral vectors deliver therapeutic genes to NPCs to: (1) upregulate COL2A1, SOX9, and EP300-SIRT5, enhancing ECM synthesis and anti-senescence; (2) silence MMPs and block IL-1B/TNF-α-NF-κB, suppressing ECM degradation and inflammation. This rebalances metabolism and delays/reverses degeneration.

Gene therapy uses a dual approach. It enhances anabolic gene expression and suppresses catabolic and inflammatory genes. RNAi can achieve specific downregulation of multiple genes in the intervertebral disc, and the CRISPR system can be used to alter the progression of intervertebral disc degeneration (Takeoka et al., 2020). Multifaceted targeting addresses the root causes of IDD. (See Figure 3 for details).

The degenerated disc’s microenvironment influences gene therapy. Hypoxia, acidity, and mechanical stress affect gene expression. Multiplex gene editing enhances cell survival in acidic conditions (Levis et al., 2023). Targeting genes that help cells adapt to stress improves outcomes.

In summary, gene therapy targets include ECM synthesis genes, matrix-degrading enzyme genes, inflammatory genes, and survival genes. Coordinating these targets restores disc homeostasis. Preclinical and clinical research will validate these targets and optimize delivery.

### 5.2 Gene delivery vectors and technologies

Gene delivery vectors are the cornerstone of effective IDD gene therapy, directly governing transfection efficiency, target gene expression kinetics, *in vivo* biosafety and final therapeutic outcomes. Unlike gene delivery in vascularized tissues, intradiscal application is uniquely constrained by the intervertebral disc’s specialized anatomy and degenerative pathological niche, which serves as the non-negotiable basis for vector selection, rather than general performance parameters alone.

Intradiscal gene delivery vector selection is guided by five core principles, all tightly linked to the disc’s inherent biological features. First, vectors must be compatible with minimally invasive fine-needle injection and maintain stable spatial retention in the disc’s closed, high-hydrostatic pressure compartment, avoiding leakage from the injection site under physiological spinal loading. Second, they need sufficient diffusivity to penetrate the disc’s dense, avascular extracellular matrix (ECM), with optimized particle size and surface charge to minimize non-specific trapping by the negatively charged proteoglycan network before reaching target nucleus pulposus (NP), annulus fibrosus (AF) or cartilage endplate (CEP) cells. Third, vectors must retain structural integrity and transfection activity in the harsh degenerative disc milieu, characterized by hypoxia, acidic pH, high osmolarity and persistent pro-inflammatory cytokines. Fourth, their immunogenicity must match the disc’s immune-privileged status; excessive immune activation not only accelerates vector clearance, but also exacerbates underlying degenerative pathology. Finally, the duration of gene expression mediated by the vector must align with therapeutic goals: transient, reversible expression for acute anti-inflammatory intervention, and stable, long-term expression for chronic ECM reconstruction in progressive IDD.

Guided by these IDD-specific principles, viral and non-viral vectors, the two core categories for intradiscal gene delivery, present distinct adaptability and application scenarios in preclinical research.

AAV6 has high *in vivo* transfection efficiency for nucleus pulposus (NP) cells (Kim et al., 2022). Host immune response is a barrier to the widespread application of AAV vectors (Arjomandnejad et al., 2023) . Pre-existing antibodies can affect the efficacy of AAV-mediated gene transfer (Verdera et al., 2020). Lentivirus-mediated fibroblast growth factor 18 gene transfer can delay intervertebral disc degeneration in rabbits (Lu and Lin, 2021). Adenoviruses have large transgene capacity, but safety issues caused by immunogenicity are a limitation of early clinical research, and they can also be used for *in vivo* gene transfer (Hackett and Crystal, 2025).

Non-viral vectors have attracted increasing attention for their low immunogenicity, high biosafety and scalable manufacturing. Polyamine-based PEG-polyplex nanomicelles delivering Runx1 mRNA can alleviate disc hydration loss in rat IDD models (Chang et al., 2022). Hydrogel-based delivery systems can be implanted into the intervertebral disc in a minimally invasive manner and achieve sustained release of gene vectors (Wang Y. et al., 2025).

Advanced delivery strategies further optimize therapeutic effects: optimized delivery of CRISPR-Cas9 components via adenoviral vectors can improve gene editing efficiency (Maggio et al., 2020). The core characteristics of various vectors are summarized in Table 2.

**TABLE 2:** Key characteristics and research applications of viral and non-viral gene delivery vectors in gene therapy for intervertebral disc degeneration.

Vector type (Category	Core performance (transfection efficiency/Duration/Immunogenicity)	Core advantages (IDD-Specific Adaptation)	Core limitations (IDD application Barriers)	IDD application scenarios	References
AAV (AAV6 preferred, Viral)	Transfection: Mod-High/Duration: Long (non-integrating)/Immunogenicity: Low	1. Low immunogenicity, preserves IDD immune-privileged status 2. Small particle size facilitates penetration of dense NP ECM 3. FDA/EMA-approved, high safety 4. Sustained low-toxicity expression in murine NP/AF cells	1. Limited capacity (single-gene only) 2. Reduced efficacy in patients with pre-existing anti-AAV antibodies	Direct intradiscal injection for murine NPCs; top candidate for clinical translation	Sun et al. (2024), Tekari et al. (2020)
Lentivirus (Viral)	Transfection: High/Duration: Long (genomic integration)/Immunogenicity: Moderate	1. Stable long-term expression, suitable for chronic progressive IDD 2. High transfection efficiency in quiescent/dividing NPCs under hyperosmolar degenerative conditions 3. Optimizable targeting to reduce off-target effects	1. Potential insertional mutagenesis risk 2. Strict dose control required for intradiscal injection	FGF-18 overexpression (anti-apoptosis, ECM restoration)	Sun et al. (2023), Tian et al. (2025)
Adenovirus (Viral)	Transfection: High/Duration: Short (rapid immune clearance)/Immunogenicity: High	1. Large capacity, easy engineering 2. Rapid high-efficiency transfection in hypoxic/acidic degenerative IDD milieu	1. High immunogenicity disrupts IDD immune homeostasis, exacerbates degeneration 2. Poor long-term *in vivo* efficacy	Early basic research; *ex vivo* cell modification only (direct injection prohibited)	Soubrier et al. (2025)
PEG-Polyamine Nanomicelles (Non-viral)	Transfection: Moderate/Duration: Short-Medium/Immunogenicity: Very Low	1. Tunable size + near-neutral charge, avoids non-specific trapping by NP ECM, enhances deep penetration 2. PEG shell protects mRNA from acid degradation	Lower transfection efficiency than viral vectors	Runx1 mRNA delivery to alleviate disc dehydration	Takeoka et al. (2020)
Hydrogel-Mediated Vectors (Non-viral)	Transfection: Moderate/Duration: Medium (sustained release)/Immunogenicity: Very Low	1. Compatible with minimally invasive fine-needle injection 2. *In situ* gelation prevents leakage under high IDD pressure 3. Isolates cargo from harsh microenvironment, controllable sustained release	1. Complex preparation process 2. Difficult to precisely regulate release kinetics	miRNA inhibitor encapsulation for IDD inflammation modulation	Tang et al. (2024)

### 5.3 Biological effects and safety of gene therapy

Gene expression regulation is vital for disc cell function. NPCs are central to disc homeostasis. Gene therapy modulates genes involved in apoptosis, ECM metabolism, inflammation, and senescence. This restores or preserves disc function.

MicroRNAs regulating apoptosis enhance NPC survival. microRNAs targeting apoptotic pathways can improve the biological function of nucleus pulposus cells and are expected to reverse IDD (Chen Y. et al., 2025). Autophagy-related genes and mTOR signaling are therapeutic targets. Hypoxia-induced autophagy via PERK signaling protects NPCs (Zhong et al., 2022) PHF6 upregulation promotes ECM breakdown in degenerative discs (Rui et al., 2020). PDGF-AB/BB downregulates senescence-associated genes. It mitigates cellular aging in human IDD cells (Zhang C. et al., 2025). Precise gene modulation restores the anabolic-catabolic balance.

Immunological response and safety are critical considerations. Viral vectors are commonly used for gene therapy, and their application safety issues and potential solutions have been discussed (Kachanov et al., 2024). The disc’s immune microenvironment includes macrophages and T cells. They produce cytokines like IL-1β and TNF-α (Chen X. et al., 2023; Ren Q. et al., 2025). Nucleus pulposus cell-specific nanoparticle carriers delivering miR-150-5p can alleviate intervertebral disc degeneration (Jiang et al., 2024). Aptamer-decorated polymeric nanoparticles deliver miRNA inhibitors. They have sustained effects in preclinical models (Jiang et al., 2024). CRISPR/Cas9 offers precise modulation but needs safety evaluation. Off-target mutations are a risk (Milheiro et al., 2025). Regulatory frameworks emphasize quality control and long-term monitoring (Jorna et al., 2021; Agrawal et al., 2025). Balancing efficacy and immune safety is a key challenge.

The degenerated disc’s microenvironment influences gene therapy. Hypoxia, nutrient deprivation, acidic pH, and mechanical stress affect outcomes. Hypoxia-induced autophagy via PERK signaling protects NPCs (Zhong et al., 2022). ASICs modulate calcium influx in acidic conditions. Inhibiting them with Sa12b improves cell proliferation (Wang Z. et al., 2022). Mechanical loading alters NPC gene expression. Mechanical loading affects the gene expression of annulus fibrosus cells, involving inflammation and osteogenesis pathways (Matyas et al., 2021; Vieira et al., 2021). Gene therapies considering these stresses have better outcomes.

In summary, gene therapy regulates gene expression to promote cell survival. It reduces senescence, modulates inflammation, and restores ECM balance. Vector immunogenicity and the disc’s inflammatory milieu are safety concerns. Advanced delivery systems and editing technologies improve specificity. Future research will refine these approaches. It will integrate disc microenvironment insights to optimize efficacy and minimize risks.

### 5.4 Clinical application prospects of gene therapy

Gene therapy for IDD has advanced in preclinical studies. It targets the molecular and cellular mechanisms of degeneration. Viral and nonviral delivery systems are delivery methods for gene therapy of intervertebral disc degeneration, and gene therapy can alter the RNA and protein synthesis of recipient cells (Takeoka et al., 2020). CRISPR-based systems can be used for gene editing research of intervertebral disc degenerative diseases (Roh et al., 2021). Autophagy is negatively regulated by mTOR and is a therapeutic target for gene therapy of intervertebral disc degeneration (Takeoka et al., 2020). Novel vectors like PCRX-201 encode IL-1Ra. They increase IL-1Ra production in degenerated NPCs. This reduces catabolic cytokines and enzymes (Snuggs et al., 2025). These findings show gene therapy can modify the disease process.

Translating to clinical practice faces challenges. The degenerated disc’s hostile microenvironment impairs delivery efficiency (Elmounedi et al., 2024; Li Z.-P. et al., 2025). Off-target effects of gene editing raise safety concerns (Milheiro et al., 2025). Advanced delivery systems address these issues. Microsphere-based carriers and multi-dynamic bond hydrogels provide sustained release (Guo et al., 2022; Chen J. et al., 2023). They protect nucleic acids and enhance cellular uptake. Selecting appropriate targets and ensuring precise regulation is key. Targeting Sox9 or ZNF865 has been investigated (Aboushaala et al., 2025; Levis et al., 2025). Molecular typing and bioinformatics enable personalized treatments (Chen et al., 2025c).

Regulatory hurdles and scalable manufacturing are additional barriers. *Ex vivo* gene therapies are approved for osteoarthritis. IDD is a potential application direction of orthopedic gene therapy (Evans et al., 2021). Safety, durability, and functional restoration need validation. Combining gene therapy with cell-based approaches or biomaterials enhances regeneration (Chen J. et al., 2023). Non-invasive or minimally invasive delivery methods facilitate clinical adoption.

In summary, gene therapy has significant potential for IDD. It addresses the root molecular causes and promotes regeneration. Advances in editing, delivery, and diagnostics overcome translation barriers. Future clinical trials will realize its potential to restore disc function.

## 6 The role of mechanobiology in the treatment of intervertebral disc degeneration

### 6.1 Mechanosensing mechanisms of intervertebral disc cells

Intervertebral disc cells endure constant mechanical forces. Pressure, tension, and shear stress come from spinal movement. These forces regulate cell behavior. They influence survival, proliferation, ECM synthesis, and inflammation.

NPCs adapt to mechanical cues. They align growth along anisotropic biomaterial microstructures. When nucleus pulposus cells are arranged and grown along the surface of MicroRods (microrods), proliferation is enhanced and apoptosis is reduced (Li Z. et al., 2025). The YAP/TAZ signaling axis maintains cytoskeletal remodeling and nuclear membrane integrity. Mechanical stress preserves cellular activity in degenerated discs.

Mechanical loading modulates cell metabolism via mechanotransduction. Fibronectin is upregulated in degenerated discs. Fibronectin activates contractility, YAP and NF-κB in nucleus pulposus cells (Naha and Driscoll, 2024). It activates cellular contractility and transcription factors like YAP and NF-κB. This suppresses proteoglycan synthesis, harming disc health.

Moderate mechanical loading activates YAP. This suppresses inflammation and promotes growth. Excessive loading inactivates YAP and activates NF-κB. 12% cyclic tensile strain can promote pro-inflammatory signaling in annulus fibrosus cells, while 5% cyclic tensile strain can inhibit inflammatory response (Wang et al., 2023c). A balanced mechanical environment is essential for disc homeostasis.

Mechanosensitive ion channels mediate responses to dynamic compression. TRPV4 activation under hyperphysiological loading regulates COX2 and PGE2. These are inflammatory mediators. Inhibiting or deleting TRPV4 reduces disc degeneration. TRPV4 links mechanical stress to inflammation.

Integrin α5β1 acts as a mechanoreceptor. It senses dynamic compressive forces in NPCs. It influences viability, apoptosis, and notochordal cell phenotype (Kanda et al., 2021). Inhibiting integrin α5β1 delays notochordal cell loss. This preserves disc cell populations during degeneration.

ECM composition and mechanical properties modulate mechanosensing. Augmented CSPG enhances anabolic turnover in NPCs. Transient receptor potential vanilloid subtype 4 (TRPV4) is activated under hydrostatic pressure (Takeoka et al., 2021). This promotes ECM synthesis and reduces fibrosis. Altered SDC4 expression under abnormal loading contributes to NP fibrosis (Sao and Risbud, 2025a; Sao and Risbud, 2025b). Sdc4 knockout mice have reduced collagen and fibronectin deposition. They preserve notochordal cell markers.

Cytoskeletal and adhesion molecules translate mechanical signals. N-cadherin (N-CDH) and integrin β1 (ITGβ1) interact on NPCs. This regulates self-renewal and ECM synthesis (Wang Y. et al., 2022). Spheroid culture enhances regenerative capacity via this axis. Moderate mechanical stimulation suppresses Cav1-mediated pro-inflammatory signaling. This reduces NF-κB activation and promotes matrix homeostasis (Zhang W. et al., 2021).

Mechanical stress influences intracellular signaling cascades. MAPK and SAPK pathways (JNK, p38) regulate apoptosis and inflammation (Cazzanelli et al., 2024; Li L. et al., 2024). MicroRNAs like miR-155-5p modulate these pathways. They enhance inflammation and catabolism under loading (Cazzanelli et al., 2024).

**FIGURE 4:**
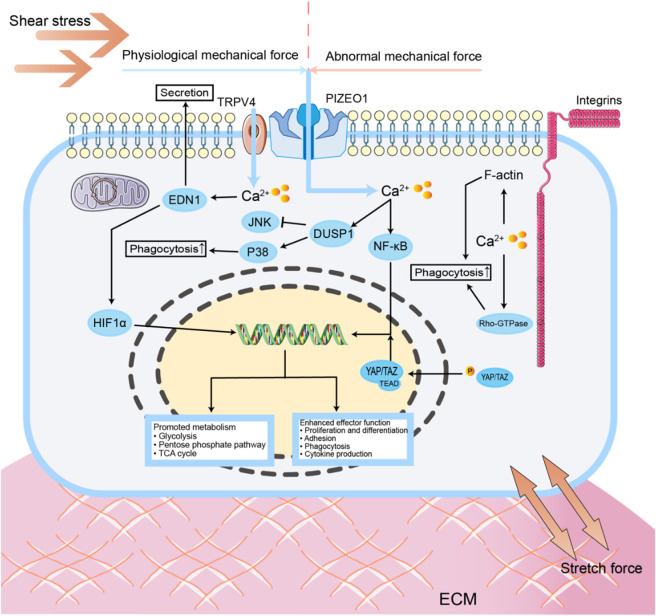
Mechanosensing and Signaling Transduction Pathways in Intervertebral Disc Cells Under Mechanical Forces: Regulatory Mechanisms of Cell Metabolism, Inflammation, and Extracellular Matrix Remodeling in Intervertebral Disc Degeneration. Anabolic axis (physiological force): Mechanical stimuli activate integrin a5ẞ1, promoting YAP/TAZ nuclear translocation and ECM component synthesis to maintain dischomeostasis. Catabolic axis (abnormal force): Excessive mechanical load activates TRPV4, triggering Ca2+ influx and NF-kB signaling, which induces pro-inflammatory cytokine (IL-1ẞ, TNF-α) release and matrix-degrading enzyme expression, driving ECM degradation and disc degeneration.

In summary, disc cell mechanosensing involves a coordinated network. ECM components, receptors, ion channels, and signaling pathways are key. They transduce mechanical forces into biochemical signals. (See Figure 4 for details). Understanding these pathways guides therapeutic strategies. Targeting mechanotransduction molecules or designing biomimetic scaffolds enhances repair.

### 6.2 Mechanical environment effects on intervertebral disc tissue engineering

Building upon the biochemical modulation strategies enabled by scaffold–growth factor combinations, the mechanical environment of the intervertebral disc constitutes an equally decisive axis in tissue engineering. Its regulatory effects not only shape the mechanical performance of scaffolds and the phenotypic behavior of disc cells but also determine whether the aforementioned regenerative signals can be effectively translated into structurally and functionally mature repair tissue.

The mechanical environment is pivotal for IDD tissue engineering. Scaffolds must match the native disc’s biomechanical properties. This provides structural support and regulates cell behavior.

Hydrogels with NP-like viscoelastic properties create an adaptive microenvironment. Self-healing injectable hydrogels can simulate the mechanical environment of natural nucleus pulposus, provide a bionic microenvironment for cells, and can be used for drug, cell delivery and tissue engineering (Gu et al., 2025). 3D-printed FPLA scaffolds have viscoelastic properties matching spine motion segments. They support NPC viability and MSC fibrocartilaginous differentiation (Marshall et al., 2021). Tailoring scaffold stiffness, elasticity, and damping is fundamental. It directly influences cell adhesion, proliferation, and ECM deposition.

Scaffold mechanical properties modulate ECM component expression. Gelatin methacrylate hydrogels of varying concentrations affect NPCs. They influence adhesion and expression of collagen types I and II and aggrecan (Li Z. et al., 2022). Mechanical cues guide stem cell differentiation. They maintain NP and AF cell phenotypes. Matching native tissue mechanics enhances cell survival. It promotes the synthesis of a functional matrix.

Dynamic mechanical loading promotes tissue formation. Bioreactor systems simulate *in vivo* motions and loads. The PRRS system cultures cell-seeded scaffolds under controlled conditions (Tekari et al., 2020). Dynamic loading enhances engineered tissue mechanical properties and composition. Mechanotransduction pathways translate mechanical stimuli into repair responses. RhoA/ROCK signaling influences AF repair (Wang Z. et al., 2023).

Mechanical stimulation modulates the inflammatory microenvironment. Adhesive hydrogels maintaining interfacial stress promote AF healing. In an annulus fibrosus repair model, the expression of COL2A1 (type II collagen encoding gene) and RhoA gene in the genipin-crosslinked Fib-T-G hydrogel combined with MSCs group is higher than that in the Fib combined with MSCs group (Wang Z. et al., 2023). These factors support matrix production and cytoskeletal organization. Combining mechanical and biochemical stimuli creates a favorable microenvironment. GDF5-loaded composite hydrogels promote the differentiation of nucleus pulposus stem cells into nucleus pulposus-like cells (Ma T. et al., 2024). This regenerates NP tissue.

In summary, the mechanical environment shapes cell behavior and tissue formation. Scaffold viscoelastic matching and dynamic loading are key. They activate mechanotransduction mechanisms for disc regeneration. Future scaffold designs will have tunable mechanical properties. This optimizes the repair of degenerated discs.

### 6.3 Mechanobiological regulation strategies

Combining mechanical stimuli with biological factors promotes disc regeneration. The IDD endures various mechanical loads. Disc cells respond dynamically to these cues. They influence ECM homeostasis.

Appropriate mechanical stimulation enhances anabolic activities. Dynamic unloading, or partial decompression, promotes water and metabolite flow. This mitigates degeneration in *ex vivo* bovine tail discs (Soubrier et al., 2025) Mechanical unloading strategies restore the disc microenvironment.

Mechanotransduction pathways convert mechanical signals into biochemical responses. Piezo1 ion channels mediate mechanical stress-induced inflammation. They trigger calcium influx, mitochondrial dysfunction, and inflammasome activation (Chen et al., 2026). This exacerbates ECM degradation and inflammation. Targeting Piezo1 modulates the mechanobiological environment. It attenuates degeneration.

The mechanical microenvironment affects NPC morphology and biophysics. TNF-α disrupts cell mechanobiology. It alters stiffness, contractility, and permeability (Hernandez et al., 2020). This depends on actomyosin contractility. RhoA activation rescues these disruptions. Mechanical forces and inflammatory signaling interact in disc pathology.

*In vitro* and organ-on-chip models recapitulate the disc’s mechanical and biochemical milieu. Flexing organ-chip devices simulate multi-directional strains on AF cells. They replicate physiological loading and enable long-term culture (McKinley et al., 2022). These platforms test mechanobiology-based therapies.

Hydrogel-based synthetic niches with tunable mechanical properties recruit stem cells. Fibronectin-modified methacrylated gelatin microspheres adjust elastic modulus and ligand density. They modulate cytoskeletal tension and YAP nuclear translocation (Chen Z. et al., 2023). This promotes NP-like differentiation without exogenous factors. Adding PDGF-BB enhances cell recruitment and matrix synthesis.

In summary, optimizing the mechanical environment with biological factors promotes IDD regeneration. Physiological mechanical loading enhances matrix synthesis and reduces inflammation. Biomaterials providing appropriate mechanical cues support cell recruitment. This integrated strategy develops effective regenerative therapies. Personalized mechanical stimulation protocols may improve outcomes. Translating these strategies to clinical practice requires further research.

### 6.4 Challenges and development directions in mechanobiology research

Replicating the disc’s physiological mechanical environment is a major challenge. The IDD endures complex, dynamic loads *in vivo*. Compression, tension, shear, and osmotic pressure vary with activity. These forces influence cell behavior and disc health.

Current *in vitro* and *ex vivo* models often fall short. Traditional 2D cultures lack 3D architecture and dynamic loading. Bioreactor-based cultures can be used for research on bovine tail intervertebral discs (Soubrier et al., 2025). But tuning mechanical parameters to mimic *in vivo* conditions is difficult. The disc’s regional heterogeneity complicates model design. NP, AF, and CEP have distinct properties. Developing region-specific, long-term culture models is essential.

Notably, goat large animal models have emerged as critical tools to bridge the ‘mechanism-translation’ gap in mechanobiology research, with studies establishing large animal models of intervertebral disc degeneration in this species (Gullbrand et al., 2024). A study on sheep spine cadaver models evaluated the effects of complex dynamic loads (axial compression combined with flexion and rotation) on L2-L3 intervertebral discs (Öztürk et al., 2024). Biomechanical and histological analyses revealed that multidimensional loads increased disc displacement and aggravated stratification deterioration of the annulus fibrosus and collagen structure damage, elucidating the mechanical mechanism of traumatic disc degeneration and guiding the development of spine implants.

Integrating mechanical regulation with molecular signaling is a key frontier. Mechanical forces are transduced via mechanosensitive molecules. Piezo1 mediates inflammation and ECM degradation (Chen et al., 2026). Actomyosin contractility modulates TNFα responses (Hernandez et al., 2020). Signaling pathways like NF-κB, MAPK, and YAP/TAZ shape cell fate. The complexity of these interactions hinders mechanistic understanding. Inflammatory microenvironments alter cell mechanobiology. This creates feedback loops that complicate research. Combining biomechanical loading with molecular techniques dissects these networks.

Advancing computational modeling and multi-scale analysis bridges mechanical and biological responses. Biphasic-swelling damage models of the AF provide insights into fluid pressure and matrix mechanics (Sun et al., 2024). They elucidate damage accumulation in degenerated discs. Computational simulations complement experimental studies. They predict tissue responses and guide scaffold design. Integrating cellular mechanobiology data with finite element models enhances understanding. Current models lack ionic transport and dynamic remodeling. Future models will be comprehensive and multi-physics-based.

Translating mechanobiological findings to clinical applications is challenging. Preclinical mechanobiology-based therapies show promise. But replicating physiological mechanical cues in humans is difficult. Device design, parameter control, and long-term assessment are barriers. IDD etiology is heterogeneous. Mechanical instability and osteoporosis contribute (Xiao et al., 2020). Advances in biomaterials offer targeted interventions (Özkale et al., 2021; Chen Z. et al., 2023). Interdisciplinary collaboration drives translation. Bioengineers, biologists, and clinicians work together to develop patient-specific therapies.

In summary, mechanobiology research faces multiple challenges. Accurate model development, integrating mechanical and molecular signaling, advancing computational models, and clinical translation are key. Innovative platforms, computational tools, and collaboration will overcome these hurdles. This advances the understanding and treatment of disc degeneration.

### 6.5 Combined therapeutic strategies

Single treatment methods (such as single cell transplantation, scaffold implantation, or biofactor delivery) have inherent defects in intervertebral disc degeneration (IDD) intervention: the hypoxic and acidic microenvironment of the degenerative intervertebral disc leads to low survival rate of transplanted cells, insufficient mechanical compatibility between the scaffold and the natural intervertebral disc, and free biofactors are easily degraded rapidly, making it impossible to achieve comprehensive and long-term IDD intervention. In contrast, combined treatment strategies integrating cells, biomaterial scaffolds, bioactive factors, and exosomes can achieve structural support, targeted cell regeneration, precise molecular regulation, and microenvironment remodeling through the synergistic effect of multiple components, becoming the core development direction of IDD regenerative therapy.

As mentioned in the previous text, the core of this cell-scaffold-biofactor ternary combination scheme is that cell therapy replenishes the functional cell pool lost due to degeneration; tissue engineering scaffolds reconstruct the natural extracellular matrix microenvironment and biomechanical support; targeted delivery systems of bioactive factors or gene carriers achieve precise molecular regulation of the degenerative microenvironment. This synergistic effect is particularly prominent in porous scaffolds: porous scaffolds can simulate the porous structure and mechanical stiffness of the natural intervertebral disc ECM, reducing cell leakage; for example, GelMA hydrogels can improve the survival rate of nucleus pulposus (NP) cells in in vitro experiments (Xu et al., 2021). Delivering growth factors such as GDF5 through such scaffolds can directed induce stem cells to differentiate into nucleus pulposus-like cells (Ma T. et al., 2024) delivering growth factors such as TGFβ3 through such scaffolds can upregulate the expression of nucleus pulposus-related genes (Kuang et al., 2021) forming a positive cycle of implantation, proliferation, and tissue regeneration. Multiple preclinical studies in animal models have also verified the efficacy of this ternary system, such as: leaf-shaped stacked structure (LSS) particles prepared from polycaprolactone and tetraethylene glycol can achieve sustained release of TGF-β3 for up to 18 days, and has stable cell adhesion (Kim et al., 2020) in another rat IDD model induced by caudal puncture, after 2 months of intervention with acellular nucleus pulposus matrix/chitosan composite hydrogel loaded with nucleus pulposus-derived stem cells (NPSCs) and GDF5-loaded microspheres, magnetic resonance imaging (MRI) analysis showed that compared with the surgical group (untreated), the intervertebral disc signal intensity of the intervention group with acellular nucleus pulposus matrix/chitosan composite hydrogel loaded with nucleus pulposus stem cells (NPSCs) and GDF5-loaded microspheres (GDF5/CH + NPSC) was significantly enhanced, suggesting that the degree of degeneration was most effectively alleviated; in a beagle IDD model, this combined system can effectively induce intervertebral disc regeneration, providing large animal experimental basis for clinical translation. Research further confirmed (Hu et al., 2020), thermosensitive hydrogels loaded with human induced pluripotent stem cells overexpressing GDF5 (GDF5-hiPSCs), after 1, 2, and 3 months of intervention in a rat IDD model, the intervertebral disc height index (DHI%) and MRI scores were significantly better than the simple puncture group and the hydrogel control group, only the voxel count of MRI-related parameters in GDF5-hiPSCs + hydrogel group was significantly lower than that in the normal group at 1, 2, and 3 months, indicating that this system can improve intervertebral disc degeneration.

Mesenchymal stem cell-derived exosomes (MSC-Exos) have emerged as a key potentiator for the tripartite system. Human urine-derived stem cell exosomes (USC-Exos) can activate the TGF-β/SMAD signaling pathway by delivering MATN3 protein, thereby promoting the proliferation of nucleus pulposus cells (NPCs) and the synthesis of extracellular matrix (ECM), while inhibiting cellular senescence. Human urine-derived stem cell exosomes have a certain improving effect on intervertebral disc degeneration in rat models (Guo et al., 2021). In addition, hypoxia-inducible factor 1α (HIF1A) can activate autophagy through the HIF1A-BNIP3-ATG7 axis, which markedly improves the survival rate of nucleus pulposus-derived stem cells (NPSCs) under mechanical compression. NPSCs with HIF1A overexpression can effectively resist overload-induced apoptosis after *in vivo* transplantation (He et al., 2021) offering a novel target for gene-modified cell combination therapy.

To further enhance therapeutic efficacy, researchers have integrated gene editing and temporal regulation technologies into the tripartite system to construct a multidimensional and precise regeneration model. Studies have shown that tonsil-derived mesenchymal stem cells engineered with a tet-off system significantly promote NPC regeneration, upregulate the expression of aggrecan and type II collagen, and simultaneously inhibit inflammatory responses and the production of pain-related neuropeptides in a rat IDD model (Kim et al., 2023). Researchers have developed esterase-responsive GHKM hydrogel microspheres that can release kartogenin (KGN) in the inflammatory microenvironment. KGN reduces reactive oxygen species production by activating the NRF2 pathway and promotes ECM synthesis in NPCs at the same time. In a rat caudal vertebrectomy model, GHKM microspheres loaded with NPCs effectively preserved intervertebral disc height and structure at 4 and 8 weeks after intervention, and histological analysis confirmed nucleus pulposus tissue regeneration (Dai et al., 2025).

Mesenchymal stem cell-derived exosomes (MSCExos) have become key enhancers of this ternary synergistic system. Their low immunogenicity, ease of loading bioactive molecules, and ability to regulate the degenerative microenvironment make them an ideal choice for combined therapy. Currently, two main application paths have been formed, both of which utilize the unique characteristics of exosomes to amplify the therapeutic effect. In exosome-stem cell synergy, urine-derived stem cell exosomes can deliver MATN3 protein to activate the TGF-β signaling pathway, promote the proliferation of nucleus pulposus cells and the synthesis of extracellular matrix (Guo et al., 2021). The anti-aging drug o-Vanillin can further promote MSCs to release exosomes; the secreted extracellular vesicles (EVs) then promote paracrine dialogue between intervertebral disc cells and MSCs, while enhancing anti-inflammatory responses and ECM synthesis (Li L. et al., 2022).

The second path integrates exosomes, stem cells, and scaffolds into a single treatment system, with the scaffold acting as a dual carrier to achieve both cell colonization and sustained exosome release. Electrospun nanofiber scaffolds have advantages in the field of intervertebral disc repair, and research progress has been made in fiber arrangement, material selection and other aspects of preparation, with clinical application prospects (Li C. et al., 2022). To further improve the therapeutic effect, researchers have integrated gene editing and mechanical regulation into the existing ternary system to construct a multi-dimensional regulation model. Using CRISPR-guided activation to regulate the ZNF865 gene that regulates ECM deposition to modify stem cells, the mechanical properties of the engineered intervertebral disc were significantly improved compared with the growth factor-treated unedited stem cell control group (Levis et al., 2025). Another study studied PCRX-201 gene therapy using human nucleus pulposus cells and tissues, and confirmed that it can continuously inhibit inflammatory responses by blocking IL1β signaling and alleviate the degree of intervertebral disc degeneration (Snuggs et al., 2025). Core-shell structured nanofiber scaffolds can achieve sequential delivery of therapeutic drugs, co-delivering the anti-inflammatory drug ibuprofen and the regeneration factor TGFβ3: ibuprofen is released rapidly to improve the inflammatory microenvironment, after which TGFβ3 is slowly released to promote extracellular matrix formation, and this has been verified in rat caudal box defect model and total disc replacement model (Han et al., 2022).

The efficacy of these combined treatment systems does not come from the simple superposition of components, but from their synergistic interaction to address the complex, multi-factor pathological nature of IDD. Despite many advances, several challenges remain to be overcome: synchronizing the release kinetics of multiple therapeutic drugs to match the intervertebral disc regeneration process; optimizing the interface compatibility between exosomes and scaffolds to improve retention and delivery efficiency; and scaling up production processes to meet GMP standards required for clinical translation. Future research may focus on developing stimulus-responsive biomaterials to achieve on-demand delivery of therapeutic components, and targeting exosome engineering to enhance specificity, making the combined strategy the most feasible path to translate preclinical success into effective clinical treatment for IDD. (See Table 3 for details).

**TABLE 3:** Multimodal synergy for IDD regenerative therapy: Cell-scaffold-biofactor integration and optimization.

Type of therapeutic strategy	Core components	Main functions and efficacy	Advantages	Limitations	References
Cell-Scaffold-Biofactor Tripartite System	Nucleus pulposus stem cells (NPSCs) + Growth differentiation factor 5 (GDF5)	In the rat tail disc puncture model, the intervertebral disc height was restored by 45% at 8 weeks, and the expression of aggrecan and type II collagen was increased by 2–3 folds compared with the single therapy group	Synergistic effect of three components: scaffold provides bionic microenvironment, GDF5 directs differentiation, significantly improving ECM synthesis efficiency	Only validated in animal models, not entered clinical stage; scaffold degradation rate needs further matching with regeneration process	Ionescu et al. (2024)
	Bone marrow mesenchymal stem cells (BMSCs) + Transforming growth factor β3 (TGF-β3)	Effectively inhibited ECM degradation in the sheep IDD model and maintained the normal mechanical function of the intervertebral disc for up to 12 months	PLGA scaffold has strong mechanical stability, TGF-β3 is continuously released, and the effect of inhibiting degeneration is long-lasting	PLGA degradation products may cause mild inflammation; size adaptation needs to be solved for translation from large animal models to clinical practice	Ashinsky et al. (2021)
	Bone marrow mesenchymal stem cells (BMSCs) + Basic fibroblast growth factor (bFGF)	Converted mechanical loads into electrical signals, regulated the NF-κB pathway to enhance ECM synthesis and inhibit inflammatory responses	The piezoelectric effect achieves “mechanical-electrical-biological” synergistic regulation without additional electrical stimulation devices	Long-term biocompatibility of PVDF materials needs to be verified; piezoelectric properties of the scaffold are easily affected by implantation position	Hao et al. (2026)
	Nucleus pulposus cells (NPCs)	The survival rate of nucleus pulposus cells was increased by more than 30% *in vitro* and *in vivo* experiments, and cell leakage was reduced	GelMA hydrogel has excellent biocompatibility and injectability, suitable for minimally invasive operations	The mechanical strength of the hydrogel is limited, and it is easy to deform under high load environments; the degradation rate is relatively fast	Li et al. (2022b)
MSC-Exos Enhanced Cell-Scaffold-Biofactor System (Exosome-stem cell-scaffold co-delivery)	Urine-derived stem cell exosomes (carrying MATN3) + Bone marrow mesenchymal stem cells (BMSCs)	Activated the TGF-β/SMAD pathway, the differentiation rate of BMSCs into NP-like cells was increased by 40%, and cell apoptosis was reduced by inhibiting caspase-3 expression	Exosomes have low immunogenicity, no risks associated with cell transplantation; MATN3 targets and activates regeneration pathways	Exosome extraction purity and yield are limited; storage and transportation conditions are harsh	Levis et al. (2025)
	Mesenchymal stem cells (MSCs) + o-Vanillin	Enhanced the release and uptake of MSC-derived exosomes, promoted paracrine crosstalk between MSCs and intervertebral disc cells, and amplified anti-inflammatory and ECM synthesis effects	o-Vanillin is easily available and low-cost; significantly improves exosome release efficiency and amplifies synergistic therapeutic effects	The optimal dosage and timing of o-Vanillin administration have not been clarified; there may be risks of non-specific cell activation	Jarrah et al. (2023)
​	Bone marrow mesenchymal stem cells (BMSCs) + MSC-Exos	In the rabbit IDD model, the intervertebral disc height retention rate reached 68% at 6 months, continuously inhibited the expression of IL-1β and TNF-α, and promoted aggrecan synthesis	Scaffold microchannels guide directional cell infiltration, exosomes continuously regulate the microenvironment; good biocompatibility	Silk fibroin processing technology is complex; the release kinetics of exosomes in the scaffold are difficult to precisely regulate	Chen et al. (2025c)
	Nucleus pulposus stem cells (NPSCs) + Exosomes	Regulated the YAP/TAZ signaling pathway to enhance directional cell arrangement and ECM deposition, and improved the structural integrity of the regenerated intervertebral disc tissue	The scaffold mimics the annulus fibrosus structure, promotes directional cell arrangement; exosomes enhance ECM deposition	The thickness of the electrospun scaffold is limited, making it difficult to adapt to severe annulus fibrosus defects; the hydrophobicity of PCL needs surface modification to improve	Han et al. (2022)
Gene Editing + Mechanical Regulation Multidimensional Combined System	CRISPR-Cas9 edited ZNF865 gene stem cells	The ECM deposition capacity of stem cells in engineered intervertebral discs was improved, and the mechanical properties of the intervertebral disc were 50% better than those of the unedited group	Gene editing precisely targets ECM deposition regulatory factors; significant improvement in mechanical properties	CRISPR-Cas9 has potential off-target risks; edited cells need to pass strict ethical reviews for clinical application	Liu et al. (2026)
	AAV6-IL-1Ra gene vector + Bone marrow mesenchymal stem cells (BMSCs)	Continuously inhibited inflammation by blocking the IL-1β signal in the mouse IDD model, the regeneration rate of nucleus pulposus cells was increased by 40%, and the severity of degeneration was reduced	AAV6 vector has high transfection efficiency and low immunogenicity; GelMA hydrogel enables co-delivery of gene vectors and cells	AAV6 vector has pre-existing immunity risks; the duration of gene expression needs to be further extended	Wang et al. (2023e)
	Ibuprofen (anti-inflammatory drug) + Transforming growth factor β3 (TGF-β3)	Rapidly cleared inflammatory factors within 72 h in the rat AF injury model, and slowly released TGF-β3 to induce stem cell differentiation, effectively reducing fibrosis and promoting annulus fibrosus repair	Core-shell structure enables sequential therapy of “rapid anti-inflammation + sustained regeneration”; the scaffold mimics the angle-ply structure of the native annulus fibrosus	The scaffold manufacturing process is complex, and large-scale production is difficult; long-term release of ibuprofen may affect cartilage metabolism	Naha and Driscoll (2024)

## 7 Animal models and current status of clinical applications

### 7.1 The role of animal models in intervertebral disc degeneration research

Animal models are indispensable for IDD research. They help investigate pathophysiology and test therapies. Various species are used. Sheep, cattle, rodents, rabbits, goats, and dogs each have unique advantages.

Large animals like sheep and cattle have disc sizes and biomechanics similar to humans. They are ideal for preclinical translation. Goat models simulate moderate-severity IDD with chondroitinase ABC. They allow assessment of intervertebral disc degeneration, endplate bone mineral density, and mechanical properties of facet joint cartilage (Zhang C. et al., 2021; Gullbrand et al., 2024). These models closely mimic human IDD features.

Rodents are widely used due to genetic manipulability and cost-effectiveness. Mice models use precise needle puncture guided by vascular anatomy. They induce lumbar IDD and replicate molecular and morphological changes (Zhang L. et al., 2022). Rat models use AF injury to induce degeneration. Behavioral assessments confirm pain analogous to humans (Lillyman et al., 2022; Yang M. et al., 2025). But their small size and disc composition differences limit direct applicability.

Rabbits balance size and experimental manageability. Their lumbar discs are accessible for surgical interventions. Needle puncture via percutaneous approach after intervertebral disc degeneration induced by puncture can reliably establish adjacent intervertebral disc degeneration model after fixation and fusion (Hei et al., 2021). They are used to model adjacent segment degeneration after fusion. This provides insights into surgical consequences (Hei et al., 2021).

Canine models are less common but useful. They enable automatic MRI grading of disc degeneration. This facilitates non-invasive evaluation of disease progression (Niemeyer et al., 2024).

Degeneration induction methods vary. Mechanical injury (needle puncture), enzymatic degradation, endplate injury, ischemia, or genetic mutations are used. Needle puncture is prevalent. Enzymatic models with chondroitinase ABC work in large animals (Gullbrand et al., 2024). Endplate injury models study the role of vertebral endplates. But none fully replicate human endplate pathology (Ma Y. et al., 2024).

Therapeutic efficacy is evaluated using multiple parameters. Histological grading, MRI, micro-CT, biochemical assays, and behavioral assessments are common. Combined therapies like hydrogel and MSC injections improve disc height in goats (Zhang C. et al., 2021). This highlights the models’ translational potential.

Model selection depends on the research question. Anatomical similarity, cost, handling ease, and ability to mimic IDD etiology are considered. No single model perfectly replicates human IDD. Integrating findings across species enhances understanding. Mechanical injury models reveal neurogenic inflammation’s role in adjacent disc degeneration (Li et al., 2024c).

In summary, diverse animal models simulate different aspects of IDD. They enable the study of molecular, cellular, and biomechanical factors. Refining and combining models improves clinical relevance. This accelerates the development of effective treatments (See Table 4 for details).

**TABLE 4:** Summary of core information on commonly used animal models for intervertebral disc degeneration (IDD).

Animal category	Core anatomical/Physiological advantages	Common modeling methods	Core limitations	References
Large animals (sheep, cattle, goats)	IDD size and biomechanical properties are close to humans, with high translational value	Enzymatic induction with chondroitinase ABC; mechanical injury (puncture/compression)	High cost, high feeding difficulty, long modeling cycle (3–12 months)	Yoshida et al. (2022), Xie et al. (2023)
Rodents (mice, rats)	Genetically editable, low cost, easy to raise, short modeling cycle	Mice: Vascular anatomy-guided lumbar puncture; Rats: Annulus fibrosus injury	Small IDD size, significant differences in composition from humans, limited translatability	Yoshida et al. (2022), Zhang et al. (2022c), Zhang et al. (2023a)
Rabbit (intermediate model)	Moderate size, easy to operate surgically, balancing controllability and relevance	Transabdominal/retroperitoneal puncture; Adjacent segment degeneration modeling after spinal fusion	Biomechanical differences from human IDDs remain	Zhang et al. (2021a)
Canine	The natural course of IDD degeneration is similar to humans	MRI-guided automatic degeneration grading modeling	High feeding cost, limited application scenarios	Zhang et al. (2024)

### 7.2 Clinical trial progress of bioengineering treatments

Biomedical engineering therapies based on cells, materials, and genes are promoting intervertebral disc degeneration (IDD) treatment to a new stage of clinical exploration from symptom relief to etiology intervention.

Biomedical engineering therapies for IDD have entered the clinical research stage, mainly focusing on cell therapy, scaffold implantation, and gene therapy. Cell therapy is currently the most active field of clinical research. An exploratory clinical trial involving 36 patients with chronic low back pain showed that intradiscal injection of autologous peripheral blood mononuclear cell-rich (PBMCs) combined with platelet-rich plasma (PRP) significantly improved patients’ pain levels and functional disability compared with the conservative treatment group during the 6-month follow-up period (Chung et al., 2024). The mechanism may be that PBMCs can differentiate into anti-inflammatory M2 macrophages, promoting extracellular matrix (ECM) repair by upregulating the expression of aggrecan and type II collagen and reducing the level of pro-inflammatory factors (Chung et al., 2024). At the same time, multiple clinical trials targeting mesenchymal stem cells (MSCs) are underway to evaluate the safety and efficacy of cell therapy, and preliminary results from MSC clinical trials have shown positive results in reducing pain and improving function (Sakai et al., 2022; Sono et al., 2024).

To overcome the problems of low survival rate and easy leakage of simple cell transplantation, scaffold materials are often used in combination with cell therapy. For example, injectable PLGA microfiber scaffolds modified with chitosan (CS) and chondroitin sulfate (CH) have been successfully used to deliver bone marrow mesenchymal stem cells (BMSCs) in large animal models. This microscaffold has potential for promoting intervertebral disc regeneration (Nakielski et al., 2023). Such biomaterials simulate the natural microenvironment and exert a synergistic effect with stem cells, significantly improving the regeneration effect (Zhao et al., 2024).

Gene therapy, as a cutting-edge strategy, shows the potential to regulate the degenerative microenvironment. An early study used genetic engineering technology to modify tonsil-derived MSCs through a tetracycline regulation system to overexpress anabolic factors such as transforming growth factor β1 (TGFβ1), insulin-like growth factor 1 (IGF1), and bone morphogenetic protein 7 (BMP7). Such engineered cells effectively promoted nucleus pulposus cell regeneration and ECM repair in preclinical models, and exhibited anti-inflammatory and analgesic effects, providing new ideas for precise treatment of IDD (Kim et al., 2023).

Precise evaluation of efficacy is the key to clinical translation. In addition to traditional pain scores and imaging indicators, functional magnetic resonance imaging techniques, such as diffusion tensor imaging (DTI) parameters (FA and Apparent Diffusion Coefficient values), have been confirmed to be closely related to biochemical changes such as GAG and collagen content in the nucleus pulposus, providing a new tool for non-invasive, quantitative evaluation of the degree of IDD degeneration (Chen et al., 2025b).

In summary, biomedical engineering therapies show great potential in IDD treatment, and are shifting from single symptom relief to a comprehensive strategy integrating cells, scaffolds, and gene regulation aimed at intervening in the degeneration process. Continuous research is expected to overcome existing problems and ultimately improve the quality of life of patients with degenerative disc disease.

### 7.3 Challenges faced in clinical applications

Despite the broad prospects, translating IDD biomedical engineering therapies from the laboratory to clinical practice still faces a series of common challenges. These challenges can be summarized as inherent biomedical problems, technology translation barriers, and disconnection from complex clinical realities.

First, immunocompatibility is the most prominent common problem. Polysaccharide-based biomaterials for intervertebral disc degeneration regenerative therapy have potential immunogenicity, and pre-existing anti-AAV antibodies can affect the efficacy of AAV-mediated gene transfer (Verdera et al., 2020; Wang X. et al., 2025). Pre-existing anti-AAV antibodies can neutralize AAV vectors and affect gene transfer efficacy, and the impact of pre-existing AAV immunity beyond neutralizing antibodies on gene transfer remains poorly understood (Verdera et al., 2020). Second, the harsh microenvironment (such as hypoxia, acidity, and inflammation) in the degenerative intervertebral disc poses severe challenges to the survival of transplanted cells, the integration of scaffolds, and the stability of gene carriers (Zhang J. et al., 2022; Li Z.-P. et al., 2025; Wang Y. et al., 2025). Inflammation is one of the pathological processes of intervertebral disc degeneration, and IL-1β and TNF-α are involved in extracellular matrix degradation and chronic inflammation in intervertebral disc degeneration (Ren Q. et al., 2025). In addition, long-term safety is an unresolved common concern, and CRISPR for intervertebral disc regeneration has off-target effects and safety and ethical challenges (Milheiro et al., 2025).

In addition to biomedical barriers, therapies face the same technical barriers when moving from laboratory research to large-scale clinical application, and these technical issues determine whether the treatment can be produced on a large scale to benefit more patients.

The primary problem is the lack of unified industry norms and standards. Currently, there are no regenerative treatment products for discogenic low back pain officially approved by the FDA (Ju et al., 2020). Consensus has not been reached on various aspects, from the optimal source and dose of MSCs, to the mechanical strength and degradation rate of scaffold materials, to the optimal vector for gene therapy, making it difficult to compare research results horizontally. Second, large-scale production of products that meet GMP standards is fraught with difficulties. Whether it is large-scale expansion of functional stem cells *in vitro*, preparation of high-precision, batch-consistent 3D printed scaffolds, or production of viral vectors with high cost and high technical thresholds, all are bottlenecks restricting industrialization. At the same time, the problems of storage and transportation of cells and biological products also need to be solved urgently.

Many technologies are effective under the ideal conditions of the laboratory, but are difficult to adapt to real clinical scenarios. For example, high mechanical pressure in the intervertebral disc leads to low cell survival rate and insufficient colonization (Dai et al., 2025); Biomaterial-based regeneration methods cannot meet the extreme mechanical requirements of the intervertebral disc (Ongini et al., 2025). The adverse microenvironment of degenerated intervertebral disc hinders the clinical application of related therapeutic methods (Elmounedi et al., 2024). More importantly, IDD itself has high disease heterogeneity and individual differences. Factors such as genetic background, age, and disease course lead to great differences in the degree of degeneration, biochemical composition, and mechanical environment of different patients, which makes the same therapy have different effects in different patients. Stem cell therapy faces challenges such as unclear survival persistence and differentiation pathways after MSCs implantation (Munda and Velnar, 2024). Mesenchymal stromal cells and their homing ability have application potential in intervertebral disc regeneration, and current treatments for intervertebral disc degeneration have limitations (Croft et al., 2021). Whether the short-term observed recovery of intervertebral disc height or pain relief can be translated into long-term functional improvement for patients for decades remains to be verified.

In summary, from laboratory discovery to wide clinical application, IDD biomedical engineering treatment still needs to cross multiple gaps such as biomedical, technical processes, and clinical adaptability. Future breakthroughs will highly depend on in-depth interdisciplinary cooperation: on the one hand, it is necessary to more deeply analyze the pathogenesis of IDD and identify more precise therapeutic targets; on the other hand, it is necessary to design a new generation of “intelligent” therapies that can dynamically adapt to complex *in vivo* environments and have both biocompatibility and mechanical stability through engineering innovation. Ultimately, promote IDD treatment to move towards individualized, standardized, and precise medicine based on molecular typing.

### 7.4 Multidisciplinary collaboration driving clinical translation

Clinical translation of bioengineering for IDD relies on multidisciplinary integration. Bioengineering, clinical medicine, and materials science are key fields. Bioengineering provides innovative tools like biomaterials and drug delivery systems. They mimic the native ECM and support regeneration. Clinical medicine contributes pathological insights and patient needs. Materials science develops biocompatible, mechanically suitable materials.

This synergy designs therapies addressing biological aspects and clinical standards. Hydrogel-based biomaterials with tunable properties are a result. They seal annular fissures and modulate inflammation post-microdiscectomy (Wang H. et al., 2025). This reduces recurrent herniation and improves outcomes. Engineering and clinical collaboration yields therapies for complex spinal pathologies.

Standardization of treatment protocols and personalized medicine accelerate translation. Standardized protocols ensure reproducibility and regulatory compliance. They enable cross-institution outcome comparison. Personalized medicine uses patient-specific data. Genetic background, degeneration stage, and biomechanics inform tailored treatments. Computational modeling simulates NPC activity under multifactorial environments (Baumgartner et al., 2021). This predicts cellular responses and guides interventions. Standardized frameworks and personalized adaptations balance applicability and optimization.

Consensus guidelines and regulatory frameworks harmonize preclinical and clinical research. Multidisciplinary expert panels emphasize clear translation pathways (Laubach et al., 2025). They address manufacturing standards, safety assessments, and ethics. Collaborative networks share knowledge and resources. This overcomes fragmented research efforts. It facilitates bench-to-bedside translation.

Emerging technologies like AI and big data enhance translational research. AI analyzes multi-omics data and patient records. It supports systems radiobiology and clinical translation (Karapiperis et al., 2021). AI-assisted biomaterial and drug delivery design accelerates development. Computational tools combined with experimental and clinical research overcome IDD treatment complexities.

Multidisciplinary education and collaborative platforms cultivate cross-field professionals. Transdisciplinary learning prepares biomedical engineers for healthcare innovation (Montesinos et al., 2023). Conferences and consortia bring together diverse expertise. They stimulate collaboration and knowledge translation (Haque et al., 2023). This ensures a sustained pipeline of clinically relevant innovations.

In summary, multidisciplinary integration drives bioengineering translation for IDD. Standardized yet personalized treatment strategies enhance efficacy. Consensus guidelines and emerging technologies support progress. Multidisciplinary education and collaboration underpin these advances. They transform experimental innovations into effective clinical interventions.

## 8 Conclusion

Bioengineering has revolutionized IDD treatment. It offers innovative strategies like cell therapy, tissue-engineered scaffolds, and gene therapy. These approaches shift from symptom management to regeneration. They target the root causes of disc degeneration.

Extensive *in vitro* and animal studies have demonstrated feasibility. Cell therapies using MSCs and NP-like cells restore cellularity and ECM composition. Biomimetic scaffolds provide structural support and cell-friendly environments. Gene therapy modulates anabolic and catabolic pathways. Mechanobiology insights optimize the mechanical environment for repair.

Despite progress, critical challenges remain. Transplanted cell survival in the harsh disc environment is low. Scaffold mechanical properties must match native tissue. Gene therapy safety and long-term efficacy need validation. Fine-tuning mechanical regulation requires deeper mechanistic understanding.

Addressing these challenges needs an integrated, multidisciplinary approach. Combining gene-modified cells with biomimetic scaffolds and mechanical stimulation enhances outcomes. Standardized preclinical models and robust outcome measures are essential.

Translating bioengineering technologies to clinical practice requires concerted efforts. Regulatory hurdles, manufacturing scalability, and cost-effectiveness must be addressed. Personalized medicine approaches tailoring treatments to patient-specific factors will improve outcomes.

In conclusion, bioengineering offers transformative potential for IDD. Progress in understanding biological and mechanical principles drives repair strategies. Multidisciplinary collaboration and iterative refinement will deliver safe, effective therapies. These therapies will address the significant unmet medical need for IDD treatment.
